# Duchenne muscular dystrophy (DMD) cardiomyocyte-secreted exosomes promote the pathogenesis of DMD-associated cardiomyopathy

**DOI:** 10.1242/dmm.045559

**Published:** 2020-11-13

**Authors:** Melanie Gartz, Chien-Wei Lin, Mark A. Sussman, Michael W. Lawlor, Jennifer L. Strande

**Affiliations:** 1 Cardiovascular Research Center, Medical College of Wisconsin, Milwaukee, WI 53226, USA; 2 Department of Cell Biology, Neurobiology and Anatomy, Medical College of Wisconsin, Milwaukee, WI 53226, USA; 3 Division of Biostatistics, Medical College of Wisconsin, Milwaukee, WI 53226, USA; 4 San Diego Heart Institute and Biology Department, San Diego State University, San Diego, CA 92182, USA; 5 Department of Pathology and Laboratory Medicine, Medical College of Wisconsin, Milwaukee, WI 53226, USA; 6 Neuroscience Research Center, Medical College of Wisconsin, Milwaukee, WI 53226, USA; 7 Department of Medicine, Cardiovascular Medicine, Medical College of Wisconsin, Milwaukee, WI 53226, USA

**Keywords:** Cardiomyocyte, Cardiomyopathy, Exosome, MicroRNA, Stress, Vesicle

## Abstract

Cardiomyopathy is a leading cause of early mortality in Duchenne muscular dystrophy (DMD). There is a need to gain a better understanding of the molecular pathogenesis for the development effective therapies. Exosomes (exo) are secreted vesicles and exert effects via their RNA, lipid and protein cargo. The role of exosomes in disease pathology is unknown. Exosomes derived from stem cells have demonstrated cardioprotection in the murine DMD heart. However, it is unknown how the disease status of the donor cell type influences exosome function. Here, we sought to determine the phenotypic responses of DMD cardiomyocytes (DMD-iCMs) after long-term exposure to DMD cardiac exosomes (DMD-exo). DMD-iCMs were vulnerable to stress, evidenced by production of reactive oxygen species, the mitochondrial membrane potential and cell death levels. Long-term exposure to non-affected exosomes (N-exo) was protective. By contrast, long-term exposure to DMD-exo was not protective, and the response to stress improved with inhibition of DMD-exo secretion *in vitro* and *in vivo*. The microRNA (miR) cargo, but not exosome surface peptides, was implicated in the pathological effects of DMD-exo. Exosomal surface profiling revealed N-exo peptides associated with PI3K-Akt signaling. Transcriptomic profiling identified unique changes with exposure to either N- or DMD-exo. Furthermore, DMD-exo miR cargo regulated injurious pathways, including p53 and TGF-beta. The findings reveal changes in exosomal cargo between healthy and diseased states, resulting in adverse outcomes. Here, DMD-exo contained miR changes, which promoted the vulnerability of DMD-iCMs to stress. Identification of these molecular changes in exosome cargo and effectual phenotypes might shed new light on processes underlying DMD cardiomyopathy.

This article has an associated First Person interview with the first author of the paper.

## Abstract (precis)

**Summary:** This study examines the consequential effects of exposure to diseased exosomes in a cell model of Duchenne muscular dystrophy (DMD). The findings support a detrimental role for long-term DMD cardiac exosome exposure.

## INTRODUCTION

Cardiomyopathy is a leading cause of death in patients with Duchenne muscular dystrophy (DMD). As an X-linked genetic disease, it is one of the most common genetic diseases worldwide, affecting ∼1:3500 to 1:5000 boys (Emery, 1991; Dooley et al., 2010). Mutations in the dystrophin gene and subsequent dystrophin deficiency lead to skeletal and cardiac muscle weakness, culminating with early mortality. Steroids and advances in respiratory therapy have increased the life expectancy for DMD by 10 years. By their late 20s and 30s, however, these patients die of complications associated with cardiomyopathy. Downstream cellular consequences of dystrophin deficiency include calcium overload, increased generation of reactive oxygen species (ROS) and mitochondrial dysfunction (Khairallah et al., 2008; Jung et al., 2008; Fanchaouy et al., 2009; Gonzalez et al., 2014). Eventually, these mechanisms culminate in cell death, leading to the muscle necrosis, inflammation and fibrosis seen in both DMD patients and animal models (Bridges, 1986; Wehling-Henricks et al., 2010; Tidball and Wehling-Henricks, 2007). Several drugs targeting these downstream mechanisms are in clinical development (Mah, 2016), but the improvements have yet to be translated into successful clinical trials. Moving forward, it will be essential to understand early molecular changes that are occurring in the cardiomyocyte in order to diagnose and treat cardiac disease effectively in DMD patients.

To gain a better understanding of the early pathological changes occurring at the molecular and cellular levels that might contribute to pathogenesis, recent interest has turned to extracellular vesicles, such as exosomes, and their potential role in cardiovascular disease (Gartz et al., 2018; Ailawadi et al., 2015). Exosomes (20-200 nm) are lipid bilayer membrane vesicles actively secreted by a variety of cells. Exosomes transfer molecular cargo, such as protein, mRNA, microRNA (miR) and lipids, from the donor cell to the recipient cell, thereby influencing the functional phenotype of the recipient cell (Cocucci et al., 2009; Kourembanas, 2015; Pap et al., 2009; Waldenström et al., 2012). Exosomes are known to change their cargo content in disease states (Gennebäck et al., 2013; Cocucci et al., 2009; Pap et al., 2009). Although exosomes represent an important mechanism for intercellular communication, little is known about the exosomal regulation of cardiomyocytes and other cells within the healthy or diseased heart.

Several investigations have focused on the therapeutic potential of exosomes for heart disease. In particular, when exosomes isolated from various progenitor or stem cells *in vitro* are injected into an injured heart *in vivo*, they display cardioprotective and restorative properties by reducing cell injury (Lai et al., 2010), diminishing hypoxia-induced inflammation (Lee et al., 2012) and transferring cardioprotective miRs into recipient cells (Ibrahim et al., 2014; Barile et al., 2014). Exosomes isolated from wild-type cardiosphere-derived cells *in vitro* and injected into DMD (*mdx*) mice *in vivo* improve cardiac function and skeletal muscle myopathy by decreasing inflammation and oxidative stress, along with improving mitochondrial function (Aminzadeh et al., 2018; Rogers et al., 2019). It is important to note that these studies used exosomes isolated from other non-endogenous cell sources. Whether endogenously secreted exosomes have a protective or pathogenic role in DMD is still under investigation. Exosomes isolated from disease-relevant models, such as diabetic cardiomyopathy or cardiac overload, induce cardiomyocyte hypertrophy (Bang et al., 2014) or impair cardiomyocyte metabolism (Bang et al., 2014; Halkein et al., 2013) via delivery of pathogenic miRs. In support, exosomes derived from DMD muscle fibroblasts were found to stimulate a profibrotic phenotype, and this was mediated by exosomal miR-199-5p (Zanotti et al., 2018). It is unknown whether similar pathogenic alterations in exosomal communication also occur in the DMD heart.

We and others have confirmed that induced pluripotent stem cell (iPSC)-derived cardiomyocytes from DMD patients are a valid model to study mechanisms and treatment approaches for DMD cardiomyopathy (Guan et al., 2014; Dick et al., 2013; Afzal et al., 2016). The purposes of this study were twofold: to elucidate the differences between healthy and diseased exosomes and to determine the mechanisms by which DMD exosomes (DMD-exo) influence the disease pathology in DMD cardiomyopathy. We hypothesize that long-term exposure to endogenously secreted DMD cardiac exosomes influences cellular phenotypes, which ultimately leads to disease pathogenesis. We recently reported that exosomes secreted from either non-affected (N) or DMD iPSC-derived cardiomyocytes (iCMs) acutely protected DMD-iCMs against the effects of metabolic stress (Gartz et al., 2018). Both exosome types were acutely protective by stimulating ERK1/2 and p38/mitogen-activated protein kinase (MAPK) signaling via an exosomal membrane surface protein. Here, a time-course study revealed that although a 2 h exposure to DMD exosomes (DMD-exo) was protective, after 24 and 48 h exposure they were no longer protective. By contrast, exposure to N-exo for 2, 24 or 48 h showed that N-exo were able to maintain their cardioprotective influence on DMD-iCMs. These findings demonstrate a role for the diverging effects of chronically diseased versus healthy exosome exposure and implicate the exosomal cargo in promoting cellular defects culminating with cardiac pathology.

## RESULTS

### The chronicity of exposure of DMD cardiomyocyte-secreted exosomes determines cytotoxic effects on DMD cardiomyocytes

We have previously shown that exposure to cardiomyocyte-secreted exosomes for 2 h protects against stress and recovery (S/R)-induced injury in DMD-iCMs by decreasing ROS levels, preserving the mitochondrial membrane potential and decreasing cell death (Gartz et al., 2018). It was uncertain whether a 2 h exposure to exosomes mimicked physiological *in vivo* conditions, where cells are continuously being exposed to secreted exosomes. Therefore, we surmised that a longer *in vitro* exposure for 24 or 48 h might provide an improved representation of *in vivo* conditions. To determine whether DMD-exo contribute to DMD cardiomyopathy, DMD-iCMs were exposed to DMD-exo at several time points to determine the effects they exerted on recipient cardiomyocytes. To do this, we used two unrelated N-iCM lines (N1 and N2), and two unrelated patient-derived DMD-iCM lines (DMD1 and DMD2), both with DMD exon 3-6 deletions, in addition to a gene-edited DMD-iCM line generated from N2 (DMDC) to serve as an isogenic control line. These cell lines were characterized previously (Gartz et al., 2018). All iCMs secreted exosomes that were similar in size and morphology and expressed typical exosomal surface markers, such as CD63 and CD81 (Fig. S1).

**Fig. 1.**
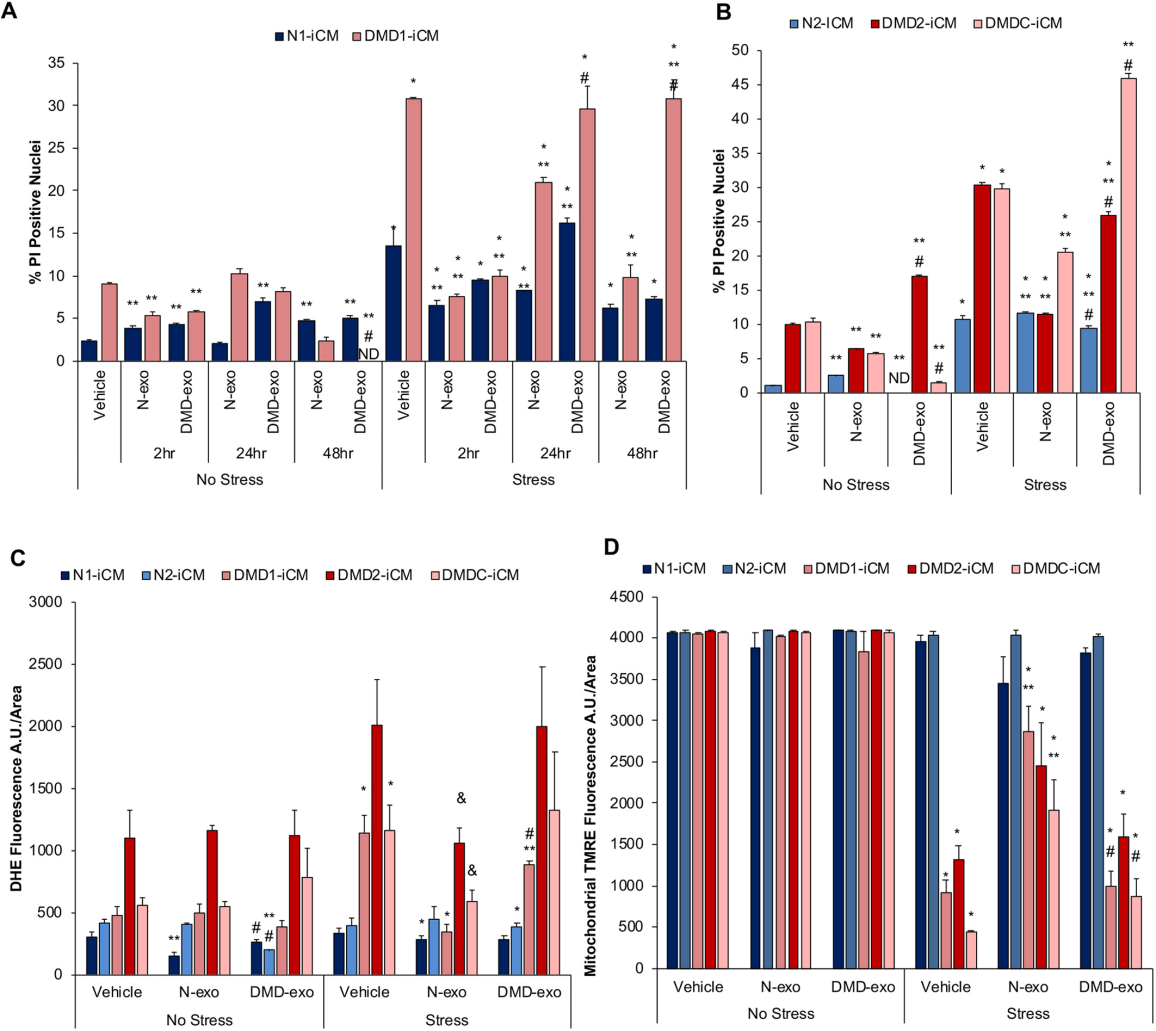
**Differential paracrine effects exerted by** **DMD cardiac exosomes** **(DMD-exo) after 2, 24 and 48 h of exposure.** Both DMD- and N-iCMs were exposed to exosomes isolated from DMD- and non-affected (N)-induced pluripotent stem cell-derived cardiomyocytes (iCMs) for 2, 24 or 48 h before stress assays. N-iCMs are represented by blue and purple bars and DMD-iCMs by red and pink bars. *n*=63-115 cells counted in each group. (A) Exposure to DMD- and N-exo for 2 h is protective, but 24 h and 48 h exposure reveals differences for DMD-exo in mitigating stress-induced cell death. *n*=91-1521 cells counted in each group. (B) Additional DMD-iCM lines demonstrate that DMD-exo no longer reduce cell death in DMD-iCMs after 48 h DMD-exo exposure. (C,D) Exposure to DMD-exo for 48 h did not reduce stress-induced ROS levels (C) or protect mitochondrial membrane potential in DMD-iCMs (D), unlike exposure to N-exo. Data represent the mean±s.e.m. Significance was determined using a two-way ANOVA. *n*=3-9 biological replicates per group; *P<0.05 stress versus no stress; **P<0.05 exosome versus vehicle; ^&^*P*=0.08 exosome versus vehicle; ^#^*P*<0.05 DMD-exo versus N-exo. A.U., arbitrary units; DHE, dihydroethidium; ND, not detected; PI, propidium iodide; TMRE, tetramethylrhodamine ethyl ester.

In preliminary time-course experiments, one N-iCM and one DMD-iCM cell line were exposed to either N- or DMD-exo for 2, 24 or 48 h, followed by 1 h of stress or control conditions and 4 h of recovery, with subsequent assessment of cell death by staining cells with propidium iodide (PI) and confirming the percentage of PI^+^ nuclei. This experiment served to indicate the effects of DMD-exo exposure on the stress response in recipient cells. Positive cardiomyocytes were selected by transduction with a lentivirus containing the cardiac marker NCX1-enhanced green fluorescent protein (eGFP), and the percentage of PI^+^ nuclei was determined through fluorescence imaging of PI-stained nuclei versus total 4′,6-diamidino-2-phenylindole (DAPI)-stained nuclei and plotted graphically. Compared with control conditions, S/R induced an increase in cell death in DMD-iCMs compared with N-iCMs in the vehicle-treated group (30±0.2% versus 14±2.1%; Fig. 1A). Both N-exo (8±0.3%) and DMD-exo (10±0.7%) exposure for 2 h before S/R protected DMD-iCMs against stress-induced cell death. Interestingly, exposure to DMD-exo for 24 h (30±2.6%) and 48 h (31±2.2) no longer reduced cell death in DMD-iCMs, whereas N-exo exposure (21±0.7%) continued to diminish cell death (Fig. 1A). Given that the differences in exosome exposure were clearly different at 48 h, we focused on this time point and validated this finding by using additional N- and DMD-iCM lines and assessing additional phenotypic responses in cardiomyocytes. In additional DMD-iCM lines, injury by S/R increased cell death in DMD-iCM groups compared with vehicle (Fig. 1B). However, exposure to N-exo, but not DMD-exo, was able to prevent cell death in DMD-iCMs (Fig. 1B). Exosomes have been reported previously to deliver dystrophin mRNA or protein transfer (Su et al., 2018). We excluded the possibility that N-exo were exerting protective effects, because 48 h N-exo exposure did not replete the dystrophin protein in DMD-iCMs (Fig. S2). A twofold increase in ROS levels after S/R was detected by dihydroethidium (DHE) fluorescence in DMD-iCMs, which was mitigated by N-exo exposure, but not DMD-exo (Fig. 1C). DHE fluorescence was detected by laser scanning confocal microscopy of DHE-stained cells, followed by image capture and analysis in ImageJ. In correlation, the mitochondrial membrane potential was decreased after S/R, as detected by loss of tetramethylrhodamine ethyl ester (TMRE) mitochondrial staining in DMD-iCMs compared with N-iCMs or control conditions. Exposure to N-exo, but not DMD-exo, preserved the mitochondrial membrane potential after S/R in DMD-iCMs (Fig. 1D). Some variability in stress response was observed among DMD-iCM lines when compared with each other (Fig. 1). In the context of this article, each cell line was compared with itself in non-stressed or vehicle conditions. These comparisons showed similar trends, indicating reproducibility (Fig. 1). These data suggest that N-exo retain their protective properties between 2 and 48 h, but this exosome-mediated protection is lost in DMD-exo over time.

### Inhibition of DMD exosome release *in vitro* and *in vivo* is associated with cardioprotection

**Fig. 2.**
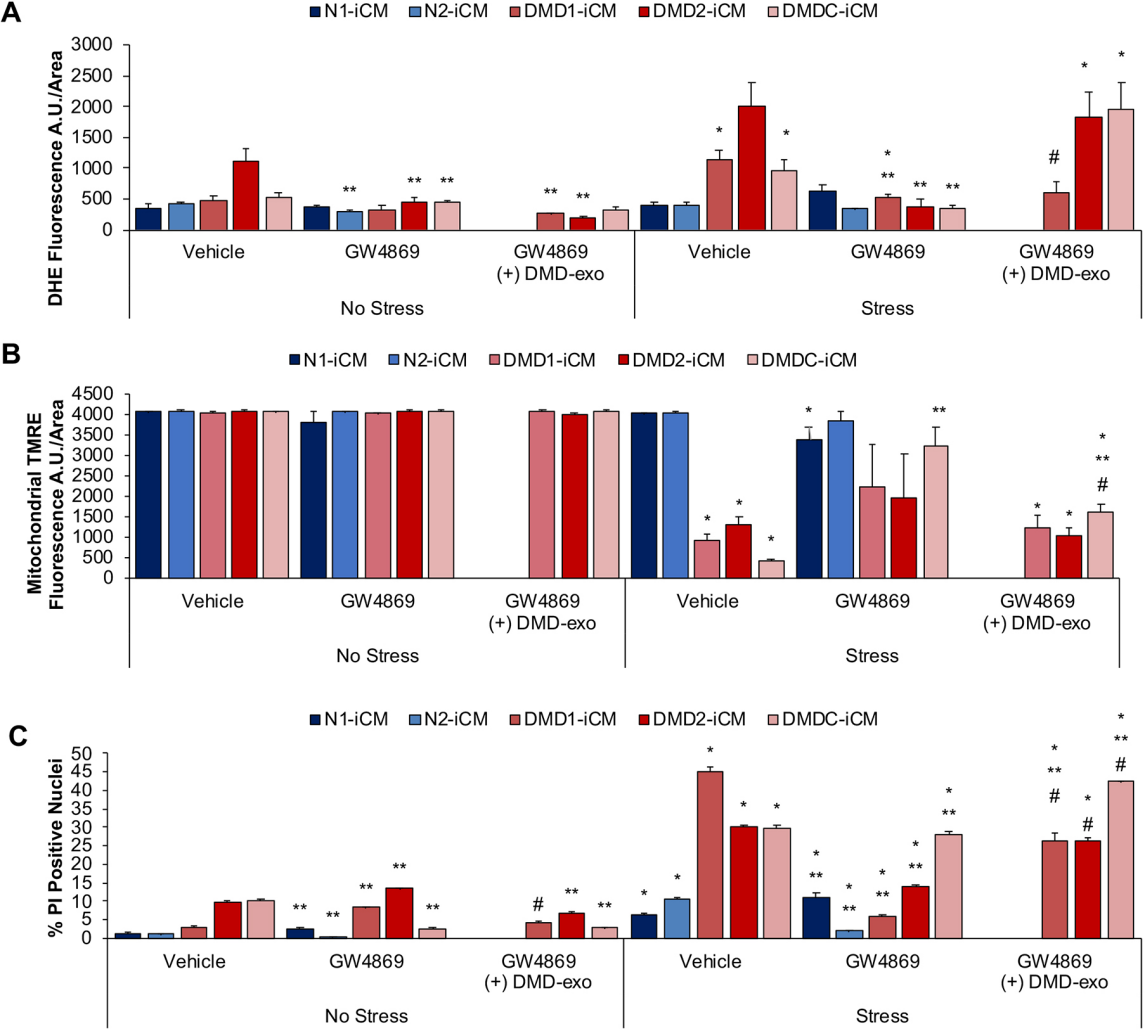
**Inhibiting DMD-exo release is cardioprotective against stress in DMD-iCMs.** Exosome release was inhibited in iCMs with 10 μM GW4869 for 24 h before stress and imaging assays. (A-C) Inhibition of DMD-exo release reduced ROS levels (A), preserved membrane potential (B) and reduced cell death (C) in DMD-iCMs. Data represent the mean±s.e.m. Significance was determined using a two-way ANOVA. In A,B, *n*=3-6 biological replicates per group; in C, *n*=52-721 cells counted in each group. *P<0.05 stress versus no stress; **P<0.05 versus (−) GW4869; ^#^*P*<0.05 GW4869+DMD-exo versus (−) GW4869.

The experiments shown in Fig. 1 suggested that long-term exposure to DMD-exo was pathogenic owing to a failure to protect DMD-iCMs from stress injury. However, it was unknown whether DMD-exo might also promote pathology, which has been described in the muscle pathology associated with DMD (Zanotti et al., 2018). To confirm whether long-term DMD-exo exposure was promoting the disease pathogenesis or merely failing to protect DMD-iCMs undergoing stress, exosome production and secretion was inhibited for 24 h with GW4869, a neutral sphingomyelinase (nSMASE) inhibitor (Trajkovic et al., 2008), followed by assessment of the functional stress response. The effective concentration and exposure time of GW4869 to inhibit exosome secretion was determined to be 10 μM for 24 h. Exposure to 10 μM GW4869 reduced N-iCM secretion from 1.59×10^8^ to 6.99×10^7^ and DMD-iCM secretion from 2.43×10^8^ to 1.28×10^8^, which were 47% and 56% reductions, respectively (Fig. S3). To verify that the effects of GW4869 were specific to its exosome inhibition activities and not attributable to off-target effects, such as ceramide reduction, DMD-exo were added along with the GW4869 treatment for 48 h. Blocking exosome secretion with GW4869 inhibited the S/R-induced increase in ROS levels in DMD-iCMs, and the addition of DMD-exo reversed the protective effects of GW4869 (Fig. 2A). Exosome inhibition also partly protected DMD-iCMs against the loss of mitochondrial membrane potential compared with the vehicle-treated group, and combining DMD-exo with the GW4869 reversed the protective effects of the compound (Fig. 2B). Likewise, GW4869 was able to limit the cell death seen in vehicle-treated DMD-iCMs, and DMD-exo reversed the protective effects of GW4869 (Fig. 2C). Together, these data show that decreasing the endogenous secretion of DMD cardiomyocyte exosomes protects against S/R-induced injury in DMD-iCMs. These results suggest not only that long-term DMD-exo exposure fails to protect DMD-iCMs from stress injury, but also that DMD-exo might have a role in promoting the disease pathogenesis of DMD cardiomyopathy.

**Fig. 3.**
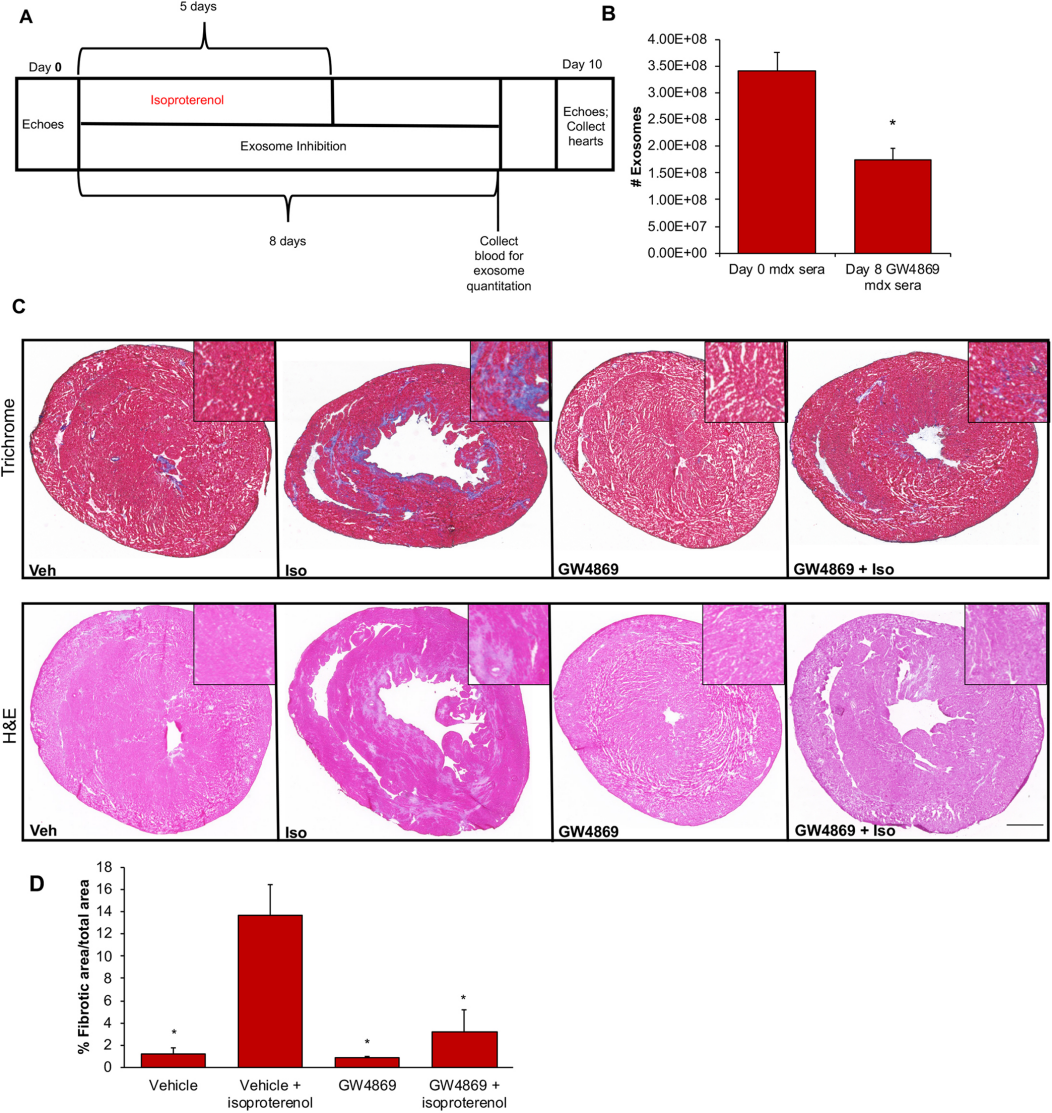
**Inhibition of** **exosome secretion in *mdx* mice protects hearts from stress-induced injury.** (A) Timeline of *in vivo* experiments in which *mdx* mice were subjected to isoproterenol-induced cardiac injury in conjunction with GW4869 exosome inhibition over 10 days, followed by histological assessment of cardiac damage. (B) Exosome quantitation assays confirm inhibition of *mdx* serum exosome levels. Data represent the mean±s.e.m. Statistical significance was determined by a one-way ANOVA. *n*=6 animals per group. *P<0.05 day 8 versus day 0. (C) Representative images from *mdx* heart sections subjected to Trichrome or Hematoxylin and Eosin (H&E) staining demonstrate isoproterenol-induced damage, which is mitigated by GW4869 exosome inhibition. (D) Quantitation of the percentage fibrotic area from H&E staining. Isoproterenol increases the percentage of cardiac fibrosis in *mdx* mouse hearts versus myocardial area, and blocking exosome secretion with GW4869 reduces fibrosis. Data represent the mean±s.e.m. Significance was determined using unpaired Student's *t*-test. *n*=6 animals per group; *P<0.05 versus vehicle+isoproterenol. Scale bar: 12.7 mm.

Next, we sought to evaluate the long-term effects of DMD-exo *in vivo*, using *mdx* mice, a well-established mouse model of DMD (Bulfield et al., 1984; Khouzami et al., 2010). Cardiac involvement in the *mdx* mouse does not occur until∼9-10 months of age (Quinlan et al., 2004; Spurney et al., 2008). Therefore, we established a short-term sub-acute injury model using 12- to 14-week-old mice to establish feasibility and potential mechanisms. For this protocol, isoproterenol [3 mg/kg/day intraperitoneally (i.p.)] was administered for 5 days to induce cardiac necrosis in *mdx* mice. Mice were treated with GW4869 (2 µg/g i.p.) or vehicle every other day, for four doses (Fig. 3A). This dosing regimen of GW4869 decreased the number of circulating exosomes from 3.41×10^8^ to 1.74×10^8^, representing a reduction of ∼49% (Fig. 3B). At the end of 10 days, ultrasound measurements and histology were performed. Assessment of cardiac function by analysis of ejection fraction, end-systolic volume and end-diastolic volume did not show any significant differences in the GW4869 treatment group (Fig. S4). However, histological analysis of *mdx* hearts revealed that GW4869 treatment reduced isoproterenol-induced cardiac fibrosis from 13.7% to 3.2% (Fig. 3C,D). Exosome inhibition in the *mdx* mouse protected the heart against isoproterenol-induced injury, thereby implicating *mdx* exosomes in promoting the cardiac pathology of DMD. Together, these data show that inhibition of DMD-exo secretion resulted in cytoprotection for DMD-iCMs (Fig. 2) and an anti-fibrotic action in stressed *mdx* mouse hearts (Fig. 3); therefore, long-term exposure to DMD diseased exosomes is implicated in the pathological processes contributing to the development of cardiomyopathy.

### The surface proteome of DMD exosomes does not contribute to the adverse long-term effects on DMD cardiomyocytes

We previously showed that exosome-associated cardioprotection at 2 h was dependent on exosomal membrane surface proteins, because exosomes lost their cardioprotective properties when their surface proteins were cleaved by trypsin (Gartz et al., 2018). Therefore, we sought to investigate whether exosomal surface proteins were involved with differential effects of long-term DMD-exo versus N-exo exposure seen after 48 h. Given that N-exo remain protective after 48 h exposure and DMD-exo are not, this suggests two possibilities: the DMD disease state promotes changes leading to either downregulation of cardioprotective proteins or upregulation of cytotoxic proteins found on the surface of DMD-exo.

**Fig. 4.**
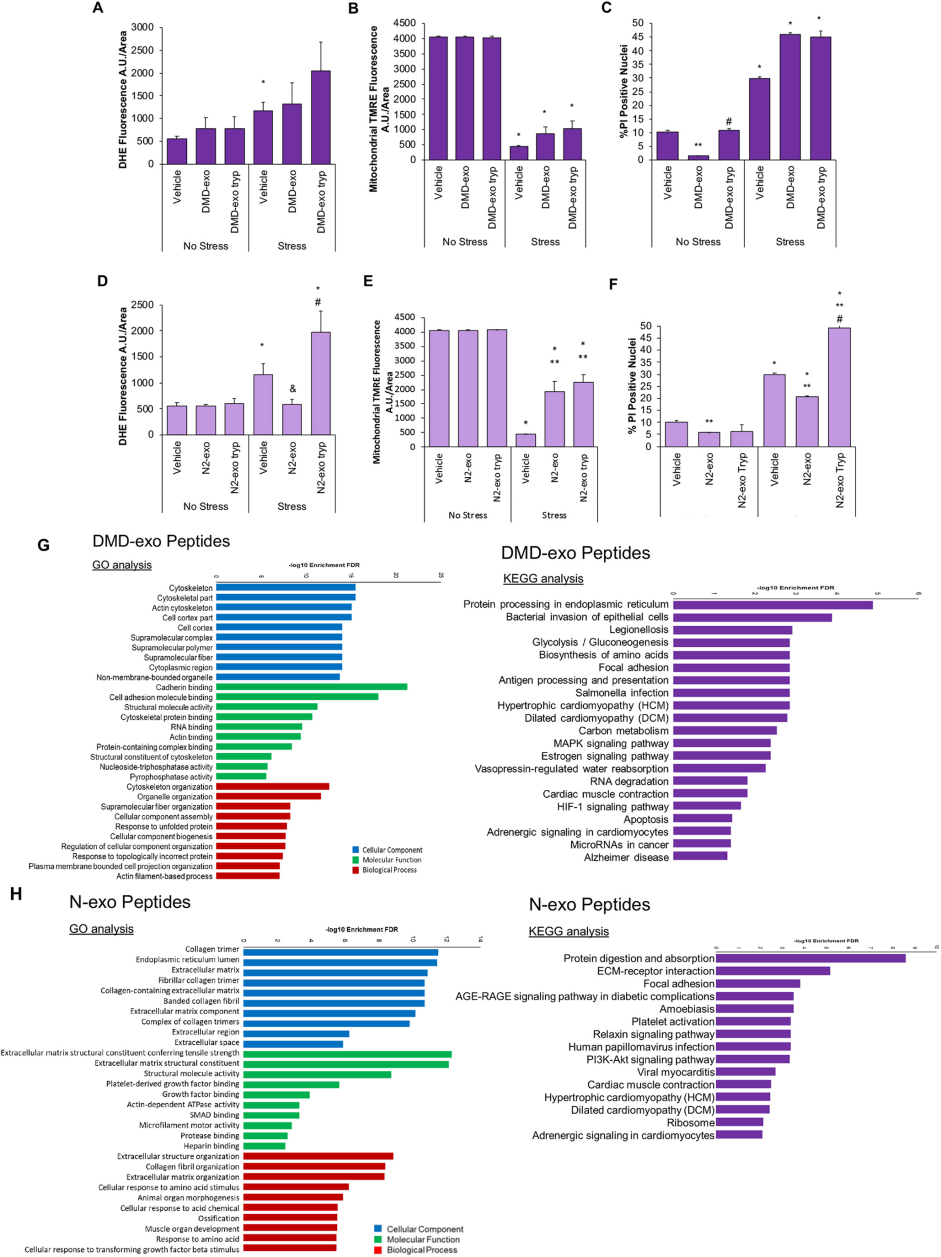
**Differences in paracrine effects exerted by long-term DMD-exo exposure are not** **attributable** **to surface peptides.** Surface peptides were stripped off exosomes with trypsin treatment, and trypsinized exosomes were used for exposure assays in DMD-iCMs. (A-C) Removal of DMD-exo surface peptides for 48 h exposure assays is associated with exacerbated stress-induced ROS levels (A), no change in mitochondrial membrane potential (B) and no alteration in cell death levels (C) in comparison to intact DMD-exo. (D-F) Trypsinization of N-exo surface peptides for exposure assays in DMD-iCMs is associated with increased stress-induced ROS levels (D), no change in mitochondrial membrane potential (E) and elevated stress-induced cell death levels (F), in comparison to intact N-exo. Data represent the mean±s.e.m. Significance was determined using a two-way ANOVA. In A,B, *n*=3-6 biological replicates per group; in C, *n*=74-165 cells counted in each group. *P<0.05 stress versus no stress; **P<0.05 exosome versus vehicle; ^#^*P*<0.05 trypsinized exosomes versus intact exosomes; ^&^*P*=0.06 trypsinized exosomes versus vehicle. Trypsinized peptides were collected from N- and DMD-exo and analyzed by mass spectrometry. (G) GO and KEGG pathway analysis of peptides expressed on DMD-exo. (H) GO and KEGG pathway of peptides expressed on N-exo. *n*=3 biological replicates per group.

For these and all subsequent studies, experiments were performed in the DMD gene-edited cell line, in order to confirm that the effects observed were attributable solely to the absence of dystrophin and not to any other differences in the genetic background of the cell line. We first determined whether the surface proteome of DMD-exo was responsible for the loss of cardioprotective effects of the DMD-exo compared with the N-exo. Cells were exposed to intact (non-trypsinized) or trypsinized exosomes for 48 h, followed by the evaluation of functional responses. Exposure of DMD-iCMs to trypsinized DMD-exo for 48 h did not reverse the S/R-induced increases in ROS levels, decreases in mitochondrial membrane potential or increases in cell death compared with intact DMD-exo (Fig. 4A-C). This indicates that the DMD exosomal surface proteome does not contain proteins that promote cytotoxicity in DMD-iCMs. Perhaps the DMD exosomal surface proteome reflects a loss of cardioprotective protein(s) that might still be present on N-exo. To address this possibility, we also analyzed the phenotypic stress response in DMD-iCMs after exposure to either trypsinized or intact N-exo. Exposure to trypsinized N-exo exacerbated S/R-induced increases in ROS levels and cell death but remained protective of mitochondria (Fig. 4D-F). In short, N-exo appear to contain a surface protein(s) that protected DMD-iCMs from S/R-induced increases in ROS levels and cell death but was not responsible for promoting preservation of the mitochondrial membrane potential. Although earlier work demonstrated that a surface peptide on both DMD-exo and N-exo was required to stimulate protection from stress after 2 h exposure (Gartz et al., 2018), studies using 48 h exposure to DMD-exo reveal that the surface peptide is no longer sufficient to stimulate protection from stress. However, long-term exposure to DMD-exo surface peptides does not appear to be associated with pathological responses to stress-induced ROS production or cell death. There might be additional effects of long-term DMD-exo cargo exposure that explain the loss of protection. In sum, these results suggest that N-exo contain surface proteins involved in protecting cells from stress-induced injury and death.

To gain a better understanding of the cytoprotective benefit of N-exo, we examined the differential expression of N- versus DMD-exosomal surface proteins using mass spectrometry analysis of peptides trypsinized from intact N- and DMD-exo (Table S1). Gene ontology (GO) and Kyoto Encyclopedia of Genes and Genomes (KEGG) pathway analyses were performed on the differentially expressed peptides. GO analysis revealed that the surface peptides preferentially detected from DMD-exo samples were enriched for proteins involved in the cytoskeletal, cell adhesion and unfolded protein response processes (Fig. 4G; Tables S2 and S3), whereas peptides preferentially detected from N-exo samples were enriched for extracellular matrix (ECM) components and growth factors (Fig. 4H; Tables S4 and S5). KEGG analysis of DMD-exo peptide samples revealed an enrichment of proteins associated with focal adhesion, MAPK signaling and adrenergic signaling, in addition to cell-injury peptides associated with hypoxia-inducible factor (HIF)-1α signaling and apoptosis (Fig. 4G). KEGG analysis of the N-exo peptides demonstrated enrichment of proteins associated with regulating protective pathways, including ECM-receptor interaction and focal adhesion, in addition to signaling through PI3K-Akt and adrenergic pathways (Fig. 4H).

Together, these analyses suggest that although the surface proteome of cardiomyocyte exosomes is altered in DMD, the sum of these changes does not alter the functional effects of long-term DMD-exo exposure in DMD-iCMs. Therefore, other changes in the molecular cargo of DMD-exo might be responsible for loss of cardioprotection on DMD-iCMs.

### The adverse functional effects of long-term exposure to DMD-exo on DMD cardiomyocytes is dependent on miR cargo

Several studies suggest that exosome-induced cardioprotection is mediated through miRs that downregulate apoptotic pathways in cardiomyocytes *in* *vitro* and in the myocardium *in vivo* (Yu et al., 2015; Wang et al., 2015, 2017; Chen et al., 2013). To confirm whether the effects of long-term DMD-exo exposure might be mediated by miR cargo, we exposed DMD-iCMs to miR-depleted exosomes, followed by assessment of the stress response. To deplete DMD-exo miR content, DMD-iCMs were treated with acriflavine, followed by harvest of exosomes for assays. Acriflavine inhibits the association of miRs with Ago2, often resulting in decreased cellular miR expression (Madsen et al., 2014; Höck and Meister, 2008; Schmidt et al., 2013). Cell treatment with acriflavine did not affect exosome secretion, because similar numbers of exosomes were detected in cells treated with or without acriflavine (Fig. S5). Treatment with 5 µM acriflavine decreased expression of cardiomyocyte and exosomal miR-101, miR-130a-3p, miR-339-5p and miR-431-5p but did not affect the levels of mRNA content, as confirmed by qPCR for Alix mRNA (Fig. S5).

**Fig. 5.**
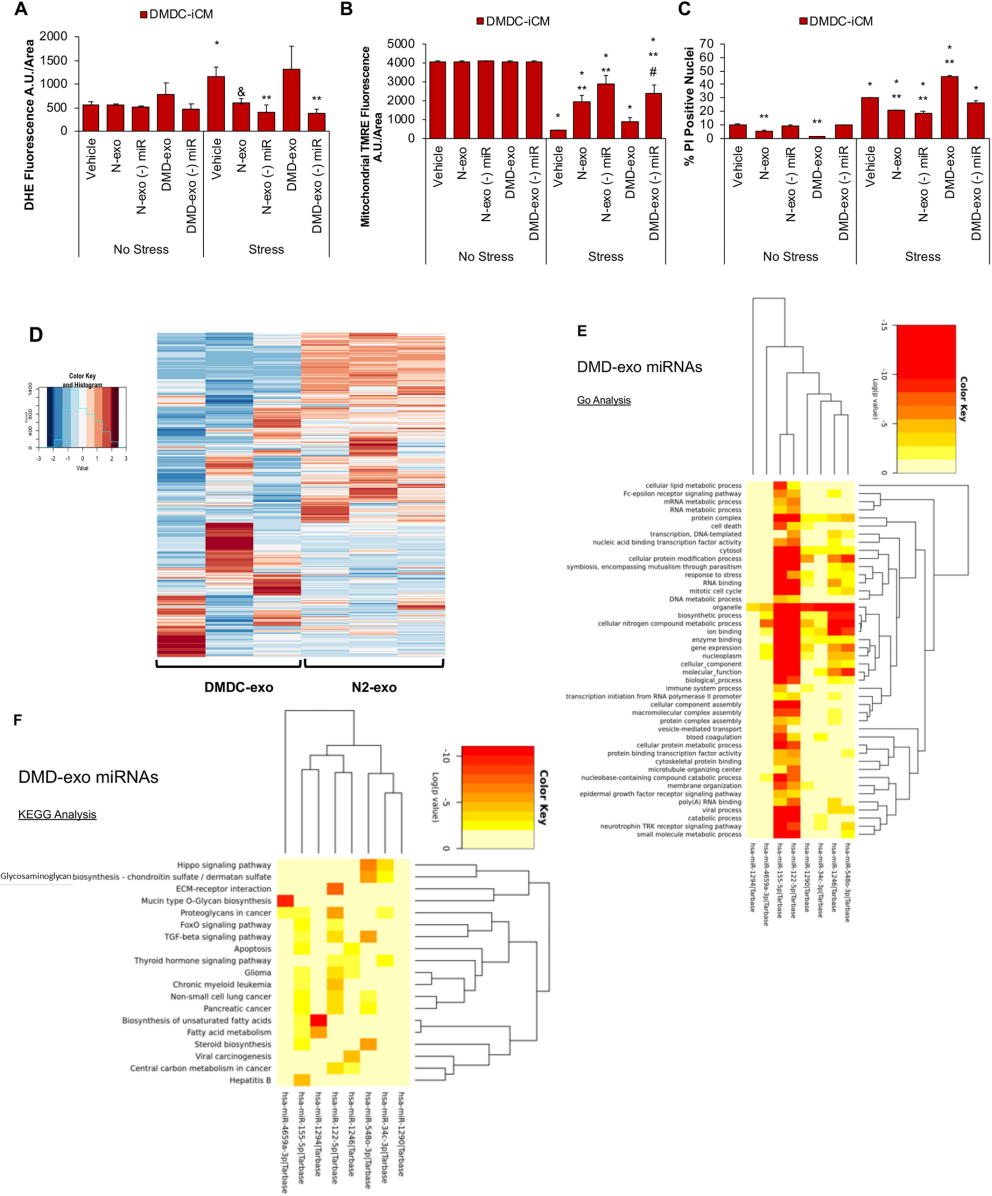
**Exosomal miR cargo contributes to altered response to stress in DMD-iCMs.** Exosomal miR cargo was depleted by exposing iCMs to 5 µM acriflavine for 2 h, followed by collection of miR-depleted exosomes for exosome exposure assays. (A) N-exo and DMD-exo depleted of miR cargo reduce stress-induced ROS in DMD-iCMs. (B) miR-depleted N-exo and DMD-exo still offer some protection against loss of membrane potential in DMD-iCMs. (C) miR-depleted N-exo and DMD-exo reduce stress-induced cell death in DMD-iCMs. Data represent the mean±s.e.m. Significance was determined using a two-way ANOVA. In A,B, *n*=3-6 biological replicates per group; in C, *n*=74-142 cells counted in each group; *P<0.05 stress versus no stress; ^&^*P*=0.07 exosome versus vehicle; **P<0.05 exosome versus vehicle; ^#^*P*<0.05 miR-depleted exosomes versus intact exosomes. (D) Hierarchical clustering of miRs identified by small RNA-sequencing in DMD-exo and N-exo reveals differential expression of 894 miRs. *n*=3 biological replicates per group. (E) GO analysis of differentially expressed DMD-exo miRs shows their involvement in regulating processes such as cell death, gene expression and cytoskeleton protein binding. (F) KEGG analysis of DMD-exo miRs highlights their regulation of pathways including apoptosis, TGF-beta signaling and Hippo signaling.

To test the functional effects of exosomal miR cargo, DMD-iCMs were exposed to miR-depleted and miR-enriched DMD-exo for 48 h and subjected to S/R injury. Compared with miR-enriched DMD-exo, miR-depleted DMD-exo mitigated the S/R-induced increase of ROS levels in DMD-iCMs (Fig. 5A). Exposure to miR-depleted and miR-enriched N-exo protected the DMD-iCMs from S/R-induced increased ROS levels. The stress-induced decrease in mitochondrial membrane potential was partly preserved by miR-enriched or depleted N-exo, in addition to miR-depleted DMD-exo (Fig. 5B). Finally, S/R increased cell death in vehicle-treated DMD-iCMs, which was exacerbated by miR-enriched DMD-exo, but not by miR-depleted DMD-exo (Fig. 5C). Exposure to both miR-enriched and miR-depleted N-exo mitigated S/R-induced cell death in DMD-iCMs (Fig. 5C). The miR cargo of DMD-exo appears to contribute to oxidative stress vulnerability, whereas N-exo protects against ROS independent of miR cargo. Together, these results suggest that the miR content of DMD-exo contributes to the adverse stress response after long-term DMD-exo exposure, whereas the N-exo miR cargo does not make a substantial contribution to DMD cardiomyocyte injury or protection and might be functionally neutral.

Given that an altered miR cargo could explain the functional differences between DMD-exo and N-exo, we then compared the miR cargo between DMD-exo and N-exo using small RNA sequencing. Hierarchical clustering was used to demonstrate the differentially expressed miRs in DMD-exo compared with N-exo (Fig. 5D). Using Qiagen IPA analysis software, the top eight DMD-exo miRs with the highest expression log ratio, in comparison to N-exo miRs, included hsa-miR-155-5p, hsa-miR-122-5p, hsa-miR-548o-3p, hsa-miR-1246, hsa-miR-1294, hsa-miR-4659a-3p, hsa-miR-1290 and hsa-miR-34c-3p. Dysregulation of these miRs has been associated with pathologies including cardiac failure, myocardial infarction, ischemia and cellular injury, such as hypoxia (Marques et al., 2016; Cortez-Dias et al., 2016; Lee et al., 2018; Li et al., 2018; Wu et al., 2017). We performed KEGG and GO enrichment analyses of genes targeted by these eight miRs. GO enrichment analysis grouped the DMD-exo miRs into several pathways involved in regulating cell death, cellular modifications, response to stress and gene expression in cardiomyocytes according to their log *P*-value (Fig. 5E). KEGG pathway analysis grouped DMD-exo miRs according to their involvement in Hippo signaling, TGF-beta signaling and apoptosis pathways (Fig. 5F). These analyses suggest unique profiles of DMD-exo versus N-exo miR content, and the altered DMD-exo miR content is associated with regulation of diverse cellular processes and pathways. These findings might help to explain the pathological effects of long-term DMD-exo exposure.

### DMD exosomal miR cargo effects transcriptional changes in DMD cardiomyocytes

**Fig. 6.**
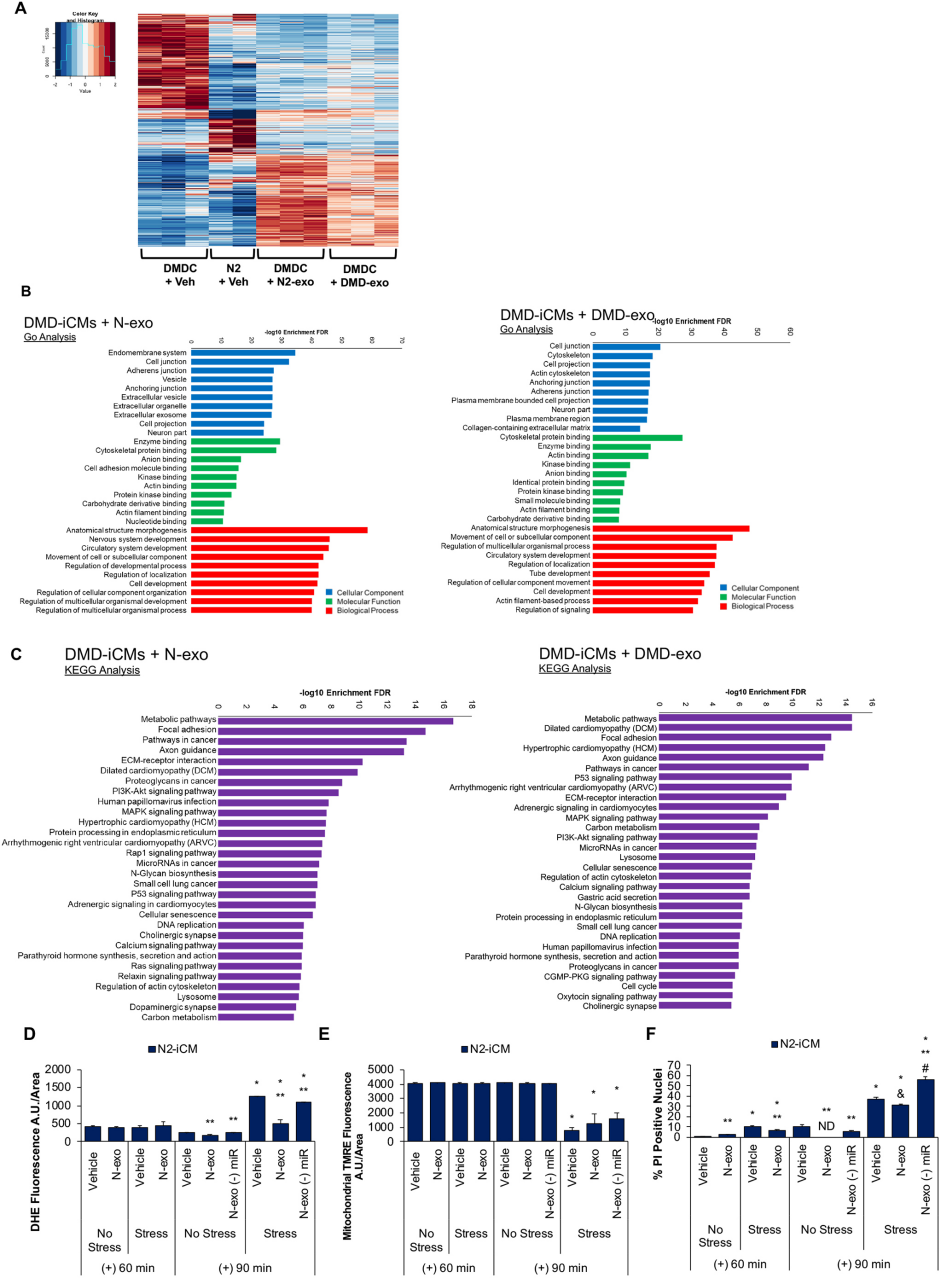
**Long-term exposure to DMD-exo leads to transcriptomic changes in DMD-iCMs** **attributable** **to altered miR cargo.** (A) Hierarchical clustering shows expression patterns of differentially expressed genes among DMD-iCMs and N-iCMs, and exposure to exosomes for 48 h leads to transcriptional alterations that are unique between DMD-exo- and N-exo-exposed groups. Red color refers to higher expression and blue color to lower expression. *n*=2-3 biological replicates per group. (B) GO analysis of DEGs in DMD-iCMs reveals that biological processes resulting from exposure to either exosome type are similar. (C) KEGG pathway analysis shows differences in pathways stimulated by N- or DMD-exo. *n*=3 per group. Next, N-iCMs were exposed to miR-depleted N-exo for 48 h before 60 or 90 min stress. (D-F) miR-depleted N-exo exacerbate ROS levels in comparison to miR-enriched N-exo (D), do not protect mitochondrial membrane potential in comparison to N-exo (E) and exacerbate cell death in N-iCMs in comparison to N-exo (F). Data represent the mean±s.e.m. Significance was determined using a two-way ANOVA. In D,E, *n*=3-6 biological replicates per group; in F, *n*=31-142 cells counted in each group. *P<0.05 stress versus no stress; ^&^*P*=0.07 exosome versus vehicle; **P<0.05 exosome versus vehicle; ^#^*P*<0.05 miR-depleted exosomes versus intact exosomes. ND, not detected.

Earlier, we demonstrated that DMD-exo miR cargo exhibited diverse effects in comparison to N-exo miR cargo, in addition to differences in the miR profile (Fig. 5). We next wanted to determine whether DMD-exo miRs promoted either the upregulation of cytotoxic pathways or the downregulation of cardioprotective pathways in DMD-iCMs. Changes in gene expression were assessed by performing RNA sequencing on DMD-iCMs before and after exposure to either N-exo or DMD-exo. Two separate expression analyses were performed using two-dimensional hierarchical clustering (Fig. 6A). First, DMD-iCMs were compared with non-affected cardiomyocytes to show baseline expression of genes without any additional treatment added. Second, DMD-iCMs treated with DMD-exo were compared with DMD-iCMs treated with N-exo to show differences in gene expression after exposure to exosome type. N-iCM+vehicle served as a control comparison group for DMD-iCM+vehicle, and DMD-iCM+N-exo served as a control comparison group for DMD-iCM+DMD-exo. Between the four groups, there were 1213 differentially expressed genes (DEGs). The transcriptome of DMD-iCMs differed from that of N-iCMs at baseline, and subsequent exposure to exosomes altered expression of numerous genes in DMD-iCMs (Tables S6-S9). Importantly, comparative transcriptional differences resulting from DMD-exo versus N-exo exposure in DMD-iCMs were observed. This suggests that paracrine effects in DMD-iCMs are impacted by the disease state of the exosome (Fig. 6A). When comparing differences in DEGs in DMD-iCMs exposed to DMD-exo versus N-exo, GO analyses of DMD-iCMs exposed to exosomes revealed that exposure to either exosome type was associated with processes such as actin binding, cytoskeletal maintenance and cell development (Fig. 6B). However, KEGG analyses revealed that exposure to N-exo altered DEGs involved in metabolism, ECM–receptor interaction and adrenergic signaling (Fig. 6C). Exposure to DMD-exo was associated with some of these same pathways; however, DMD-exo exposure was uniquely associated with cGMP-PKG and TGF-beta pathways, which are known to be defective and increased, respectively, in DMD (Shirokova and Niggli, 2013; Khairallah et al., 2008; Cohn et al., 2007).

Given that there are DEGs between DMD-iCMs and N-iCMs at baseline (Fig. 6A), we asked whether the baseline transcriptome of the donor cell determines the effectual phenotype exerted by the exosomes. Our standard S/R injury protocol does not induce injury in N-iCMs; therefore, in order to induce a stress response in N-iCMs, cells were subjected to 90 min stress and 4 h recovery. Stress-induced ROS levels in N-iCMs were reduced by miR-enriched N-exo, but only minimally by miR-depleted N-exo (Fig. 6D). Neither the miR-enriched nor the miR-depleted N-exo were able to preserve the mitochondrial membrane potential in N-iCMs (Fig. 6E). Cell death was slightly reduced by miR-enriched N-exo, but exacerbated by miR-depleted N-exo (Fig. 6F).

In sum, these data suggest that the stress response of the cell is affected by the disease status of the cell and whether it has been exposed to healthy or diseased exosomes. When considering the effects of DMD-iCMs exposed to DMD-exo and N-iCMs exposed to N-exo, it appears that DMD-exo promote the S/R injury response via miR cargo. This injury response might be attributable to its alteration of gene expression, as suggested by Fig. 6A. In support, KEGG pathway analyses demonstrated that exposure to DMD-exo versus N-exo differentially impacted signaling through important cytotoxic and protective pathways (Fig. 6C), which further implicates DMD-exo and their cargo in cellular deficiencies.

## DISCUSSION

The major findings of this study are that DMD-exo exert varied functional effects on recipient cells depending upon the duration of exposure and the underlying disease state of the recipient cell type. More specifically, the paracrine effects of DMD exosome exposure shift from protective to pathogenic over longer periods of exposure. The 2 h exposure period reveals the acute effects of exosomes on iCMs, whereas the 24-48 h exposure period reveals the chronic effects of exosomes on iCMs. Non-diseased, N-exos maintained their protective properties over DMD-iCMs throughout the acute and chronic phases of the study and depended on a surface protein to exert these effects. However, exposure to chronically diseased DMD-exo promoted S/R injury through changes to the miR cargo. Some phenotypic variability was observed among the three DMD-iCM lines. However, statistical comparisons were performed within each cell line compared with control conditions. These comparisons were fairly consistent, suggestive of a stable phenomenon. In corollary experiments, exosome inhibition with GW4869 was used to block endogenous exosomal paracrine effects. Exosome inhibition *in vitro* abolished S/R-induced injury in DMD-iCMs. Likewise, exosome inhibition *in vivo* significantly reduced isoproterenol-induced cardiac injury in *mdx* mice. In summary, these findings suggest that DMD-exo promote injury in DMD-iCMs and might contribute to the disease pathogenesis.

The results of this study support our initial hypothesis that DMD-exo would be functionally detrimental to DMD-iCMs by exacerbating S/R-induced injury. Previously, we reported that the effects of N-exo and DMD-exo were both functionally protective after an acute 2 h exposure in DMD-iCMs, which failed to support this initial hypothesis (Gartz et al., 2018). Acute effects of exosomes were independent of the donor cell disease state, because both N-exos and DMD-exos had similar cardioprotective effects. The effects of acute versus chronic exosome exposure had not previously been defined completely in the literature. The present study clarifies acute effects after 2 h exosome exposure and chronic effects after 24-48 h exosome exposure. Long-term exposure to exosomes reveals diverging effects stimulated by DMD-exo versus N-exo, because DMD-exo fail to protect cells from S/R injury, whereas N-exo stimulate cardioprotection. Circumstances known to influence the effects exerted by exosomes include the disease status of the donor cell, exosome composition and even dosage (Davidson et al., 2018; Tabak et al., 2018; Gartz and Strande, 2018). Here, we show that the duration of exposure to diseased exosomes is another factor to consider when examining the paracrine effects they stimulate.

Exosome inhibition with GW4869, an inhibitor of nSMASE, was cardioprotective *in vitro* and *in vivo*. Inhibition of nSMASE-2 results in the decreased synthesis of ceramides, and through this mechanism, GW4869 has been reported to be cardioprotective (Liao et al., 2013; Pavoine and Pecker, 2009). Ceramide accumulation has been associated with mitochondrial dysfunction and apoptosis (Andrieu-Abadie et al., 2001; Yu et al., 2007), which are stress-induced phenotypes observed in DMD-iCMs. We did not measure ceramide levels in our study to verify that our findings were not attributable to a reduction in ceramide. Although this might be a limitation to our study, several previously published studies have attributed the effects of GW4869 to its ability to inhibit exosome release (Essandoh et al., 2015; Wang et al., 2014a; Lyu et al., 2015; Menck et al., 2017). In the present study, we verified that exosome inhibition was the mechanism by which GW4869 was acting to promote cardioprotection by adding isolated DMD-exos with the GW4869. The addition of isolated DMD-exos and GW4869 reversed the effects seen with GW4869 alone, suggesting that the mechanism is specific to exosome inhibition effects of the compound, rather than other mechanisms of GW4869.

Endogenously secreted exosomes have been demonstrated to be involved in the pathogenesis of diseases including diabetic cardiomyopathy (Wang et al., 2014b), cancer (Corcoran et al., 2012; Demory Beckler et al., 2013) and DMD skeletal myopathy (Zanotti et al., 2018). Here, we demonstrate that DMD cardiac exosomes contribute to pathophysiological changes in DMD cardiomyocytes. The cargo and secretion of exosomes change in conditions of stress (Guo et al., 2017; Németh et al., 2017; de Jong et al., 2012). In the context of neuromuscular diseases, such as DMD, it is unknown how the molecular cargo of a secreted exosome shifts from protective to pathogenic, thereby leading to dysregulated cellular responses.

To define the divergent effects between N-exo and DMD-exo resulting from long-term exposure, we first examined the potential role of the surface proteome of DMD-exo. Exosomal surface peptides have been reported to stimulate paracrine effects in destination cells (Gartz et al., 2018; Vicencio et al., 2015; Gastpar et al., 2005; Gupta and Knowlton, 2007; Emmanouilidou et al., 2010), largely by stimulating the signaling pathways in target cells, ranging from protective (Gartz et al., 2018; Vicencio et al., 2015) to pathogenic (Webber et al., 2010). Our previous study shows that the exosome-mediated cardioprotection that results from acute (2 h) exposure is dependent on exosomal surface proteins, regardless of whether the exosomes are derived from diseased or healthy cardiomyocytes. However, upon longer periods (24-48 h) of exosome exposure, the exosome-mediated effects of diseased DMD-exos shift from protective to pathological. By contrast, N-exos maintain their cardioprotective properties, regardless of the duration of exposure. Exosomal surface peptides do not appear to be involved with the pathogenic stress response, because exposure to trypsinized DMD-exo produces similar phenotypic responses to intact DMD-exo exposure. Previously, we showed that both DMD and N-exo stimulated cardioprotection through ERK1/2 and MAPK signaling (Gartz et al., 2018). Although we did not examine whether long-term exosome exposure still stimulated either of these pathways, comparative analyses of the exosome surface proteome revealed that DMD-exo contained peptides associated with protective signaling, such as MAPK and adrenergic signaling, in addition to peptides associated with cell injury pathways, such as apoptosis and HIF-1α. Cardioprotective signaling through MAPK has been reported to be stimulated by exosomes in DMD cardiomyopathy (Gartz et al., 2018) and ischemic injury to the myocardium (Vicencio et al., 2015). Likewise, impaired adrenergic signaling and aberrant levels of apoptosis have been shown to underlie cardiac dysfunction in models of DMD cardiomyopathy (Li et al., 2014; Lin et al., 2015; Xi et al., 2000). Here, KEGG analysis of the surface proteome of DMD-exo identified peptides associated with stimulation of MAPK signaling (Fig. 4). Given that long-term exposure to DMD-exo led to pathogenic effects, perhaps long-term exposure to the injury-associated surface peptides or other exosome cargo might explain the loss of cardioprotection. The surface proteome of N-exo primarily contained peptides associated with protection and survival, such as those within the PI3K-Akt pathway, a well-known survival pathway in cardiomyocytes (Dhanasekaran et al., 2008; Lin et al., 2015; Kuwahara et al., 2000). Of note, PI3K-Akt has previously been reported as impaired in DMD cardiomyopathy (Peter and Crosbie, 2006); therefore, it is possible that N-exo might promote some protective benefits of the PI3K-Akt pathway in DMD-iCMs. The finding that N-exo exert cardioprotection through surface proteins has important implications for other studies investigating the potential benefit of healthy cell-derived exosomes in mediating cardiac repair (Vicencio et al., 2015). Identification of the proteins that might be absent on DMD-exo, yet present on N-exo, might provide key information for future studies to examine such candidates for cardioprotection.

Multiple studies have shown that the miR cargo of exosomes isolated from a variety of cellular sources was responsible for beneficial cellular effects (Yu et al., 2015; Wang et al., 2017; Zhang et al., 2017). However, endogenously secreted exosomes have been adversely implicated in cardiovascular and neuromuscular diseases (Grad et al., 2010; Rajendran et al., 2006; Zanotti et al., 2018; Bang et al., 2014; Halkein et al., 2013). Notably, exosomal miRs have been implicated in the skeletal myopathy associated with DMD (Zanotti et al., 2018). We used acriflavine to decrease miR expression levels. Acriflavine is an inhibitor of Ago2, therefore blocking the loading of miRs to the RNA-induced silencing complex (RISC) complex. Studies have shown that acriflavine is effective at decreasing cellular miR levels (Madsen et al., 2014; Lauschke et al., 2016), but it is unknown how it impairs miR loading into exosomes. Here, we show that the loss of protective benefit of DMD-exo exposure was attributable to miR cargo, because exposure to miR-depleted DMD-exo reversed these effects. The loss of benefit might also be associated with the absence of cardioprotective signaling or uptake that we previously found with acute DMD-exo exposure (Gartz et al., 2018), although these possibilities were not examined in the scope of this project. Closer examination of DMD exosomal miR cargo by small RNA sequencing confirmed differential patterns of expression in comparison to N-exo. These differences in exosomal miR expression might explain the divergent effects of healthy versus DMD-exo, because altered miR cargo derived from diseased exosomes is known to exert transcriptional effects (Zanotti et al., 2018; Singh et al., 2014). Exposure to exosomes had effects on gene transcription, because transcriptomic analysis of DMD-iCMs exposed to DMD-exo revealed differential expression of genes associated with cellular pathways and processes such as cGMP-PKG, p53 signaling and TGF-beta signaling. Disruption of these pathways has previously been observed in cell and animal models of DMD cardiomyopathy (Afzal et al., 2016; Lin et al., 2015; Shirokova and Niggli, 2013; Mourkioti et al., 2013). Increased TGF-beta signaling has been observed in DMD cardiomyopathy (Cohn et al., 2007) and is known to promote cardiac fibrosis (Lijnen et al., 2000). Blocking DMD-exo release *in vivo* was associated with reduced cardiac fibrosis (Fig. 3), and this might be attributable to reduced chronicity of exposure to DMD-exo and their miR cargo. Overall, these data suggest that the miR cargo of DMD-exo promotes transcriptional changes that might influence disease pathogenesis in DMD.

We now provide evidence that long-term exposure to DMD diseased exosomes promotes pathophysiological cellular changes. Exosome inhibition mitigated pathological processes associated with DMD cardiomyopathy in an *mdx* mouse model. Most exosome-related cardiac therapies are focused on the delivery of exosomes. However, exosome inhibition strategies represent a paradigm shift in the treatment of myocardial disease. Furthermore, these findings identify a temporal shift in the cellular response to exosomes secreted from diseased cardiomyocytes in an *in vitro* model of DMD cardiomyopathy and might have implications for other cardiomyopathies. Going forward, it will be important to examine exosome cargo closely in various disease conditions in parallel with examination of molecular changes in the cell at the same time points. This will give greater understanding of the changes that exosomes are exerting in nearby and distant cells during disease processes. Better understanding of these discrete changes might lead to more highly specialized treatments and preventive strategies and lead to better tools for clinicians to track the progression of cardiomyopathy in DMD.

## MATERIALS AND METHODS

### Cell lines, iPSC culture and cardiac differentiation

In this study, we used five previously characterized iPSC lines: two unrelated, non-affected patient-derived lines, N1 and N2, and two unrelated, dystrophin-deficient patient-derived lines, DMD1 and DMD2 (Gartz et al., 2018); a third dystrophin-deficient line, DMDC, was generated by CRISPR-Cas9 gene editing from N2 (Gartz et al., 2018). All cell lines were maintained on Matrigel (Corning, NY, USA)-coated plates with Mtesr1 (Stem Cell Technologies) media. Mycoplasma contamination is tested for every 6 months using the Venor GEM mycoplasma detection kit (Sigma-Aldrich) according to the manufacturer's protocol. Cardiac differentiations were performed as previously described (Gartz et al., 2018). In brief, iPSCs were dissociated with gentle dissociation reagent (Thermo Fisher Scientific, Bremen, Germany) and plated at a density of 1,000,000 cells per well in a Matrigel-coated 12-well plate. On day −1, all cell lines were overlaid with Matrigel in Mtesr1 media. On day 0, N1, DMD1 and DMD2 received 12 µM CHIR-99021 (CHIR; Selleck Chem) in a Matrigel overlay with Roswell Park Memorial Institute (RPMi) medium 1640 without insulin (Thermo Fisher Scientific); N2 and DMDC received 10 µM CHIR and 10 ng/µl Activin A (R&D Systems). On day 1, the medium was replaced on all cell lines with RPMi medium 1640 without insulin. On day 3, the medium was replaced on all cell lines with RPMi medium 1640 without insulin containing 10 µM inhibitors of Wnt production 2 (IWP2; Stemgent). On day 5, the medium was replaced on all cell lines with RPMi medium 1640 without insulin, and on day 7 and beyond, all cell lines were maintained in RPMi 1640+insulin medium. Cells contracted as early as day 9 and were used in assays after 30 days of contracting.

### Cell dissociation for assays

Cardiomyocytes contracting for 30±5 days were dissociated as previously described (Gartz et al., 2018). Cardiomyocytes were trypsinized with 0.05% trypsin (Thermo Fisher Scientific) for 5 min, and the reaction was inactivated with Dulbecco's modified Eagle's medium (Thermo Fisher Scientific) containing 10% fetal bovine serum (Thermo Fisher Scientific). Cells were seeded onto Matrigel-coated glass coverslips for imaging at a density of 50,000 cells per coverslip.

### Live cell and cell imaging assays

For live cell imaging analyses of iCMs, cells were transduced with an NCX1-eGFP lentiviral construct 3-4 days before imaging (Ovchinnikov et al., 2015). This construct encodes an enhanced green fluorescent protein under the control of the cardiac-specific promotor NCX1, therefore identifying living iCMs for imaging and subsequent image analysis. Lentiviral vector assembly and titer were performed by Vector and Molecular Biology Core Lab at the Blood Research Institute (Milwaukee, WI, USA). To detect ROS levels, iCMs were stained with 10 µM DHE (Sigma-Aldrich) for 20 min, followed by washout with RPMi+insulin maintenance medium. To detect mitochondrial membrane potential, iCMs were stained with 50 nM TMRE for 20 min. Images were taken by confocal fluorescent microscopy (Nikon A1-R) using NIS Elements software (v.5.11.00, 64 bit), and the intensity of fluorescence of DHE and TMRE was detected at excitation/emission wavelengths of 518/605 nm or 540/595 nm, respectively. Nuclei were counterstained with Hoechst 33342 (excitation/emission 350/461 nm). Imaging conditions were held constant during assays. Image analysis was performed using ImageJ software (v.1.52p, Java 1.8.0_172; National Institutes of Health). For analysis, five randomly selected regions of interest were selected from nuclei (DHE) or mitochondria (TMRE) and measured for the mean intensity of fluorescence.

### Exosome isolation

Cells were plated 200,000 cells per well in a 12-well plate, and medium was conditioned for 48 h, followed by harvesting for exosome isolation. Medium was collected from each well and used for separate exosome preparations. Exosomes were isolated using the Total Exosome Isolation Reagent from Cell Culture Media (Thermo Fisher Scientific) according to the manufacturer's instructions. Exosome pellets were resuspended in 100 µl of 0.2 µM filtered PBS and stored in small aliquots to avoid freeze-thaw cycles. In select assays, cells were treated with 10 µM acriflavine (Sigma-Aldrich) and the medium was conditioned for 2 h, followed by harvest of the medium and exosome isolation. Exosomes were harvested from *mdx* mouse serum using the Total Exosome Isolation Reagent from Serum (Thermo Fisher Scientific) according to the manufacturer's instructions.

### Exosome characterization

Isolated exosomes were characterized by transmission electron microscopy as previously described (Gartz et al., 2018). In brief, exosomes were adsorbed on freshly ionized, 400 mesh formvar/carbon grids, then washed with distilled water and negatively stained with 2% aqueous uranyl acetate. Exosomes were viewed with a Hitachi H600 transmission electron microscope and imaged with a Hamamatsu CCD camera with AMT imaging software. The surface proteins CD63 and CD81 were analyzed using Exo-ELISA-Ultra detection kits (System Biosciences) according to the manufacturer's protocol. Exosome surface peptides CD63, CD81, ALIX, FLOT1, ICAM1, EpCam, ANXA5 and TSG101 were also characterized using the Exo-Check Exosome Antibody Array (System Biosciences) according to the manufacturer's instructions.

### Exosome exposure and stress injury protocol

Forty-eight hours before cell stress assays, 5 µl of resuspended exosomes were added to iCMs. The cells were washed with PBS to remove endogenous exosomes, and resuspended exosomes were added in medium to each well to represent consistent, basal exosome levels across all cells. In select experiments, 5 µl of resuspended exosomes was added to cells 2 or 24 h before stress. In each experiment, one preparation of exosomes was used. Cells on coverslips were exposed to stress injury as described previously (Gartz et al., 2018). In brief, cells received 60 min exposure to 100 µM H_2_O_2_ in 10 mM deoxyglucose in RPMi without glucose (Thermo Fisher Scientific), followed by 4 h recovery in maintenance medium. In select experiments on N-iCMs, N-iCMs were exposed to stress for 90 min, followed by 4 h recovery in maintenance medium.

### Fixed cell imaging (cell death) assays

For PI cell death staining assays, iCMs were fixed with 4% paraformaldehyde (Alfa Aesar) 24 h after stress injury and stained with PI (Thermo Fisher Scientific) according to the manufacturer's protocol, followed by mounting onto glass slides with Fluoroshield containing DAPI (Sigma-Aldrich). Images of fixed cells were taken by laser scanning confocal microscopy. Images were analyzed in ImageJ for the percentage of PI^+^ nuclei versus total nuclei. In these experiments, *n* is the total number of nuclei imaged and analyzed.

### Trypsinization of exosomes and mass spectrometry for exosome peptides

Exosomes were collected from 1×10^6^ DMD2- or N1-iCMs, in triplicate, and the exosome pellet was subjected to five cycles of centrifugation at 10,000 ***g*** and washing with 0.22 µM filtered PBS to prepare samples for mass spectrometry. Next, isolated exosomes were trypsinized as described previously (Gartz et al., 2018), and trypsinized peptides were collected and processed by the Medical Genome Facility, Proteomics Core at the Mayo Clinic (Rochester, MN, USA). Dried trypsin-digested samples were suspended in 0.2% formic acid, 0.1% trifluoroacetic acid and 0.002% Zwittergent 3-16. A portion of the sample was analyzed by nano-flow liquid chromatography electrospray tandem mass spectrometry (nanoLC-ESI-MS/MS) using a Thermo Scientific Q-Exactive Mass Spectrometer (Thermo Fisher Scientific) coupled to a Thermo Ultimate 3000 RSLCnano HPLC system. The digested peptide mixture was loaded onto a 330 nl Halo 2.7 ES-C18 trap (Optimize Technologies). Chromatography was performed using a 5-45% gradient of solvent B for 90 min (where solvent A is 98% water, 2% acetonitrile and 0.2% formic acid, and solvent B is 80% acetonitrile, 10% isopropanol, 10% water and 0.2% formic acid). Peptides were eluted at a flow rate of 400 nl/min from the trap through a PicoFrit (New Objective) 100 µm×33 cm column hand packed with Agilent Poroshell 120 EC C18 packing (Agilent Technologies). A Q-Exactive mass spectrometer was set to acquire ms1 survey scans from 340 m/z to 1500 m/z at a resolution of 70,000 (at 200 m/z) with an automatic gain control (AGC) target of 1×10^6^ ions and a maximum ion injection time of 50 ms. Survey scans were followed by higher-energy collisional dissociation tandem mass spectrometry (HCD MS/MS) scans on the top 15 ions at a resolution of 17,500, AGC target of 1×10^5^ ions, maximum ion inject time of 75 ms, and the isolation window set at 3.0 m/z, with a 0.5 m/z offset. Dynamic exclusion placed selected ions on an exclusion list for 30 s.

### Analysis of mass spectrometry results

MaxQuant software (Max Planck Institute of Biochemistry), v.1.6.0.16, was used to extract, time align and database search chromatographically extracted peptide peaks generated from mass spectrometry files (Cox and Mann, 2008; Tyanova et al., 2016a). Label-free relative quantitation parameters within the MaxQuant software were used to generate normalized protein intensities reported in a protein groups table (Cox et al., 2014). Perseus software, v.1.6.2.1 (Max Planck Institute of Biochemistry) was used to perform differential expression of identified proteins (Tyanova et al., 2016b). Briefly, protein intensities were log2 transformed; Student's *t*-tests were performed, in which an estimation of difference was calculated, and *P*-values and q-values were reported. Protein groups shown as either present/absent or a q-value of ≤0.05 were considered to exhibit a significant differential expression and selected for further analysis. GO and KEGG pathway analysis of peptides using ShinyGO v.0.61 analysis software was performed using a false discovery rate (FDR) cut-off of 0.05, and the top 10 most enriched GO cellular components, molecular functions and biological processes were shown, along with the top 30 most significantly enriched KEGG pathways.

### RNA extraction and qPCR

RNA was isolated from iCMs using the Purelink RNA mini kit (Thermo Fisher Scientific) according to the manufacturer's instructions. RNA was isolated from exosomes using the Total Exosome RNA and Protein Isolation kit (Thermo Fisher Scientific) according to the manufacturer's instructions. RNA was resuspended in RNAse-free water and quantified on a Nanodrop 2000 Spectrophotometer (Thermo Fisher Scientific). Reverse transcription of iCM RNA was performed using the iScript cDNA synthesis kit (Bio-Rad), and the reaction included 5 min at 25°C, 20 min at 46°C, 1 min at 95°C and holding at 4°C. qPCR was performed using the SsoAdvanced Universal SYBR Green Supermix (Bio-Rad), and the reactions were carried out with an initial step of 30 s at 95°C, followed by 40 cycles of denaturation (15 s at 95°C) and annealing (30 s at 60°C), followed by fluorescence capture and holding at 4°C. Primers used included ALIX (forward: 5′-GACGCTCCTGAGATATTATGATCAGA-3′ and reverse: 5′-ACACACAGCTCTTTTCATATCCTAAGC-3′). Data were analyzed using the ΔΔ*Ct* method in CFX Manager software (Bio-Rad), and the RPLP0 Quantitect Primer Set (Qiagen) was used as a normalization control.

Reverse transcription of miRNA was performed with the miScript RT II kit, according to the manufacturer's instructions for mature miRNAs, on a Bio-Rad C1000 Touch thermal cycler. qPCR for RNU6 and hsa-miR-130a was performed using the miScript SYBR Green PCR Kit (Qiagen), which involved an initial activation step of 15 min at 95°C, followed by 40 cycles of denaturation (15 s at 94°C), annealing (30 s at 55°C) and extension (30 s at 70°C), followed by fluorescence data capture and holding at 4°C. qPCR was performed on a Bio-Rad CFX96 Real Time System. Primers for RNU6 and hsa-miR-130a were commercially designed by Qiagen. Data were analyzed using the ΔΔ*Ct* method and CFX Manager software, and RNU6 served as a normalization control.

### RNA sequencing and analysis

For RNA-sequencing of iCMs, 1 µg of RNA was harvested from N2 or DMDC-iCMs, with or without 48 h exosome exposure, and sent to BGI Genomics for library construction and 30 most significantly enriched reads on BGISeq500 50SE. Samples were harvested in biological duplicate or triplicate to represent *n*=2 or 3. Preprocessing of FASTQ files was performed by BGI Genomics. Gene counts are summarized from BAM files by the software program featureCounts (v.1.6.2) (Liao et al., 2014). The parameters used were as follows: number of threads used, 10; chimeric fragments, not counted; multi-mapping reads, counted; feature type in the annotation file, exon; fragments counted instead of reads; attribute type used to group features, gene_id. Gene counts were normalized and used to compare various group differences with statistical software R package ‘edgeR’ (Robinson et al., 2010). For each gene in a group comparison, a *P*-value and FDR (by the Benjamini–Hochberg procedure; Benjamini and Hochberg, 1995) are given for the downstream selection of markers. Genes identified by differential expression analysis (FDR<0.05) were used to generate a heatmap using the R package ‘gplots’ and function ‘heatmap.2’, with a dendrogram showing co-regulation structure among genes (Fig. 6A). GO and KEGG pathway enrichment analysis of differentially expressed genes was performed using ShinyGO v.0.61 (Ge and Jung, 2018 preprint). Given the FDR cut-off of 0.05, the top 10 most enriched GO cellular components, molecular functions and biological processes are shown in Fig. 6B,C, along with the top 30 enriched KEGG pathways.

For small RNA-sequencing of exosomal miRNA, 1 µg of RNA was harvested from N2 or DMDC-iCMs and sent to BGI Genomics for small RNA library construction and 30 most significantly enriched reads on BGISeq500 50SE. Preprocessing of the data and statistical analysis were performed by BGI Genomics. Differentially expressed exosomal miRs were identified (FDR<0.05 and a log fold change <−0.5 or >2) and used to generate a heatmap as described above (Fig. 5D). GO and KEGG pathway enrichment analysis was performed using miRPath v.3 (Vlachos, et al., 2012) for the differentially expressed miRNAs, shown as heatmaps in Fig. 5E,F. Samples were harvested in biological triplicate to represent *n*=3.

### Animal studies

Animal studies were approved by the Institutional Animal Care and Use Committee at the Medical College of Wisconsin (approval no. #3547). All mice in this study were 10- to 12-week-old female C57BL/10ScSn-Dmdmdx/J mice (The Jackson Laboratory). All animals were handled in compliance with Institutional Animal Care and Use Committee guidelines as well as local, national and international regulations and guidelines.

### GW4869 exosome inhibition *in vitro* and *in vivo*

For exosome inhibitor experiments in iCMs, 10 µM GW4869 (Selleck Chem) was added to the cell culture medium 24 h before stress assays. For exosome inhibition in *mdx* mice, 12-week-old *mdx* mice received i.p. injections of 2 μg/g GW4869 every other day on days 0-8 and isoproterenol (3 mg/kg) daily from day 2 to 6. Blood was collected on day 8 for serum exosome quantitation. To quantify exosomes, the exosome protein concentration was first determined by the BCA assay (Thermo Fisher Scientific) and standardized to 50 μg for performing quantitation via the Exo-cet Exosome Quantitation Kit (System Biosciences) according to the manufacturer's instructions. Data were analyzed relative to the exosome standard included in the kit.

### Histological analysis of *mdx* hearts

Hearts from *mdx* mice were harvested and flash frozen in liquid nitrogen. Cryostat sections were cut at 8 µM thick for staining. Histological staining for H&E was performed at the Children's Hospital of Wisconsin Research Institute's Histology Core (Medical College of Wisconsin). Trichrome staining was performed using the Surgipath Gomori Trichrome Staining kit (Leica) according to the manufacturer's instructions. Multiple sections were analyzed in different planes, and representative images are shown in Fig. 3. Imaging was performed on a Nikon Super Coolscan 9000 using VueScan ×64 v.9.6.06, and images were analyzed in MetaMorph Software (Molecular Devices), taking into account the percentage fibrotic area versus the total area of the heart section.

### Immunofluorescence for dystrophin

Cardiomyocytes (N or DMD) were fixed and stained for dystrophin using NCL-DysB (Leica Novocastra, NCL-DYSB, batch 6055813) as described previously (Gartz et al., 2018). Briefly, iCMs were treated with or without N-exo for 48 h, followed by washing with PBS and fixing with 4% paraformaldehyde for 15 min at room temperature. Cells were exposed to 1:50 NCL-DysB and 1:500 Alexa Fluor 488 goat anti-mouse antibody (Invitrogen, A-21131), followed by mounting coverslips on slides with Fluoroshield containing DAPI (Sigma-Aldrich) and imaged by laser-scanning confocal microscopy. Three coverslips were imaged, with a total of ten fields viewed per coverslip.

### Echocardiography of *mdx* hearts

Cardiac function of *mdx* mice was assessed by the Echocardiography Core (Medical College of Wisconsin) on days 0 and 10 of the GW4869 exosome inhibition protocol. The percentage ejection fraction and end-systolic volume data were analyzed. All *mdx* mouse experiments were performed with *n*=6 animals per group.

### Statistical analysis

All *in vitro* experiments were performed in biological triplicate (*n*=3) per experimental group and condition, unless otherwise noted. Experimental results are presented as the mean±s.e.m. Statistical analyses, including Student's *t*-test and one-way ANOVAs, were performed as appropriate, using GraphPad Prism software, and significance was determined as *P*≤0.05.

## Supplementary Material

Supplementary information

## References

[DMM045559C1] Afzal M. Z., Reiter M., Gastonguay C., Mcgivern J. V., Guan X., Ge Z.-D., Mack D. L., Childers M. K., Ebert A. D. and Strande J. L. (2016). Nicorandil, a nitric oxide donor and ATP-sensitive potassium channel opener, protects against dystrophin-deficient cardiomyopathy. *J. Cardiovasc. Pharmacol. Ther.* 21, 549 10.1177/1074248416636477 26940570 PMC5010518

[DMM045559C2] Ailawadi S., Wang X., Gu H. and Fan G.-C. (2015). Pathologic function and therapeutic potential of exosomes in cardiovascular disease. *Biochim Biophys Acta* 1852, 1-11. 10.1016/j.bbadis.2014.10.008 25463630 PMC4268281

[DMM045559C3] Aminzadeh M. A., Rogers R. G., Fournier M., Tobin R. E., Guan X., Childers M. K., Andres A. M., Taylor D. J., Ibrahim A., Ding X. et al. (2018). Exosome-mediated benefits of cell therapy in mouse and human models of duchenne muscular dystrophy. *Stem Cell Rep.* 10, 942-955. 10.1016/j.stemcr.2018.01.023 PMC5918344 29478899

[DMM045559C4] Andrieu-Abadie N., Gouazé V., Salvayre R. and Levade T. (2001). Ceramide in apoptosis signaling: relationship with oxidative stress. *Free Radic. Biol. Med.* 31, 717-728. 10.1016/S0891-5849(01)00655-4 11557309

[DMM045559C5] Bang C., Batkai S., Dangwal S., Gupta S. K., Foinquinos A., Holzmann A., Just A., Remke J., Zimmer K., Zeug A. et al. (2014). Cardiac fibroblast-derived microRNA passenger strand-enriched exosomes mediate cardiomyocyte hypertrophy. *J. Clin. Invest.* 124, 2136-2146. 10.1172/JCI70577 24743145 PMC4001534

[DMM045559C6] Barile L., Lionetti V., Cervio E., Matteucci M., Gherghiceanu M., Popescu L. M., Torre T., Siclari F., Moccetti T. and Vassalli G. (2014). Extracellular vesicles from human cardiac progenitor cells inhibit cardiomyocyte apoptosis and improve cardiac function after myocardial infarction. *Cardiovasc. Res.* 103, 530-541. 10.1093/cvr/cvu167 25016614

[DMM045559C7] Benjamini Y. and Hochberg Y. (1995). Controlling the false discovery rate: a practical and powerful approach to multiple testing. *J. R. Stat. Soc. B* 57, 289-300. 10.1111/j.2517-6161.1995.tb02031.x

[DMM045559C8] Bridges L. R. (1986). The association of cardiac muscle necrosis and inflammation with the degenerative and persistent myopathy of MDX mice. *J. Neurol. Sci.* 72, 147-157. 10.1016/0022-510X(86)90003-1 3711930

[DMM045559C9] Bulfield G., Siller W. G., Wight P. A. and Moore K. J. (1984). X chromosome-linked muscular dystrophy (mdx) in the mouse. *Proc. Natl Acad. Sci. USA* 81, 1189-1192. 10.1073/pnas.81.4.1189 6583703 PMC344791

[DMM045559C10] Chen L., Wang Y., Pan Y., Zhang L., Shen C., Qin G., Ashraf M., Weintraub N., Ma G. and Tang Y. (2013). Cardiac progenitor-derived exosomes protect ischemic myocardium from acute ischemia/reperfusion injury. *Biochem. Biophys. Res. Commun.* 431, 566-571. 10.1016/j.bbrc.2013.01.015 23318173 PMC3732190

[DMM045559C11] Cocucci E., Racchetti G. and Meldolesi J. (2009). Shedding microvesicles: artefacts no more. *Trends Cell Biol.* 19, 43-51. 10.1016/j.tcb.2008.11.003 19144520

[DMM045559C12] Cohn R. D., Van Erp C., Habashi J. P., Soleimani A. A., Klein E. C., Lisi M. T., Gamradt M., Ap Rhys C. M., Holm T. M., Loeys B. L. et al. (2007). Angiotensin II type 1 receptor blockade attenuates TGF-β-induced failure of muscle regeneration in multiple myopathic states. *Nat. Med.* 13, 204-210. 10.1038/nm1536 17237794 PMC3138130

[DMM045559C13] Corcoran C., Rani S., O'Brien K., O'Neill A., Prencipe M., Sheikh R., Webb G., McDermott R., Watson W., Crown J. et al. (2012). Docetaxel-resistance in prostate cancer: evaluating associated phenotypic changes and potential for resistance transfer via exosomes. *PLoS ONE* 12, e50999 10.1371/journal.pone.0050999 PMC3519481 23251413

[DMM045559C14] Cortez-Dias N., Costa M. C., Carrilho-Ferreira P., Silva D., Jorge C., Calisto C., Pessoa T., Robalo Martins S., De Sousa J. C., Da Silva P. C. et al. (2016). Circulating miR-122-5p/miR-133b ratio is a specific early prognostic biomarker in acute myocardial infarction. *Circ. J.* 80, 2183-2191. 10.1253/circj.CJ-16-0568 27593229

[DMM045559C15] Cox J. and Mann M. (2008). MaxQuant enables high peptide identification rates, individualized p.p.b.-range mass accuracies and proteome-wide protein quantification. *Nat. Biotechnol.* 26, 1367-1372. 10.1038/nbt.1511 19029910

[DMM045559C16] Cox J., Hein M. Y., Luber C. A., Paron I., Nagaraj N. and Mann M. (2014). Accurate proteome-wide label-free quantification by delayed normalization and maximal peptide ratio extraction, termed MaxLFQ. *Mol. Cell. Proteomics* 13, 2513-2526. 10.1074/mcp.M113.031591 24942700 PMC4159666

[DMM045559C17] Davidson S. M., Riquelme J. A., Takov K., Vicencio J. M., Boi-Doku C., Khoo V., Doreth C., Radenkovic D., Lavandero S. and Yellon D. M. (2018). Cardioprotection mediated by exosomes is impaired in the setting of type II diabetes but can be rescued by the use of non-diabetic exosomes in vitro. *J. Cell Mol. Med.* 22, 141-151. 10.1111/jcmm.13302 28840975 PMC5742744

[DMM045559C18] de Jong O. G., Verhaar M. C., Chen Y., Vader P., Gremmels H., Posthuma G., Schiffelers R. M., Gucek M. and Van Balkom B. W. M. (2012). Cellular stress conditions are reflected in the protein and RNA content of endothelial cell-derived exosomes. *The J. Extracell. Vesicles* 1 10.3402/jev.v1i0.18396 PMC3760650 24009886

[DMM045559C19] Demory Beckler M., Higginbotham J. N., Franklin J. L., Ham A.-J., Halvey P. J., Imasuen I. E., Whitwell C., Li M., Liebler D. C. and Coffey R. J. (2013). Proteomic analysis of exosomes from mutant KRAS colon cancer cells identifies intercellular transfer of mutant KRAS. *Mol. Cell. Proteomics* 12, 343-355. 10.1074/mcp.M112.022806 23161513 PMC3567858

[DMM045559C20] Dhanasekaran A., Gruenloh S. K., Buonaccorsi J. N., Zhang R., Gross G. J., Falck J. R., Patel P. K., Jacobs E. R. and Medhora M. (2008). Multiple antiapoptotic targets of the PI3K/Akt survival pathway are activated by epoxyeicosatrienoic acids to protect cardiomyocytes from hypoxia/anoxia. *Am. J. Physiol. Heart Circ. Physiol.* 294, H724-H735. 10.1152/ajpheart.00979.2007 18055514 PMC2443685

[DMM045559C21] Dick E., Kalra S., Anderson D., George V., Ritso M., Laval S. H., Barresi R., Aartsma-Rus A., Lochmüller H. and Denning C. (2013). Exon skipping and gene transfer restore dystrophin expression in human induced pluripotent stem cells-cardiomyocytes harboring DMD mutations. *Stem Cells Dev.* 22, 2714-2724. 10.1089/scd.2013.0135 23829870 PMC3787465

[DMM045559C22] Dooley J., Gordon K. E., Dodds L. and Macsween J. (2010). Duchenne muscular dystrophy: a 30-year population-based incidence study. *Clin. Pediatr.* 49, 177-179. 10.1177/0009922809347777 20080524

[DMM045559C23] Emery A. E. H. (1991). Population frequencies of inherited neuromuscular diseases—a world survey. *Neuromuscul. Disord.* 1, 19-29. 10.1016/0960-8966(91)90039-U 1822774

[DMM045559C24] Emmanouilidou E., Melachroinou K., Roumeliotis T., Garbis S. D., Ntzouni M., Margaritis L. H., Stefanis L. and Vekrellis K. (2010). Cell-produced alpha-synuclein is secreted in a calcium-dependent manner by exosomes and impacts neuronal survival. *J. Neurosci.* 30, 6838-6851. 10.1523/JNEUROSCI.5699-09.2010 20484626 PMC3842464

[DMM045559C25] Essandoh K., Yang L., Wang X., Huang W., Qin D., Hao J., Wang Y., Zingarelli B., Peng T. and Fan G.-C. (2015). Blockade of exosome generation with GW4869 dampens the sepsis-induced inflammation and cardiac dysfunction. *Biochim. Biophys. Acta* 1852, 2362-2371. 10.1016/j.bbadis.2015.08.010 26300484 PMC4581992

[DMM045559C26] Fanchaouy M., Polakova E., Jung C., Ogrodnik J., Shirokova N. and Niggli E. (2009). Pathways of abnormal stress-induced Ca2+ influx into dystrophic mdx cardiomyocytes. *Cell Calcium* 46, 114-121. 10.1016/j.ceca.2009.06.002 19604578 PMC2745084

[DMM045559C27] Gartz M. and Strande J. L. (2018). Examining the paracrine effects of exosomes in cardiovascular disease and repair. *J. Am. Heart Assoc.* 7, e007954 10.1161/JAHA.117.007954 29858362 PMC6015376

[DMM045559C28] Gartz M., Darlington A., Afzal M. Z. and Strande J. L. (2018). Exosomes exert cardioprotection in dystrophin-deficient cardiomyocytes via ERK1/2-p38/MAPK signaling. *Sci. Rep.* 8, 16519 10.1038/s41598-018-34879-6 30410044 PMC6224575

[DMM045559C29] Gastpar R., Gehrmann M., Bausero M. A., Asea A., Gross C., Schroeder J. A. and Multhoff G. (2005). Heat shock protein 70 surface-positive tumor exosomes stimulate migratory and cytolytic activity of natural killer cells. *Cancer Res.* 65, 5238-5247. 10.1158/0008-5472.CAN-04-3804 15958569 PMC1785299

[DMM045559C30] Ge S. and Jung D. (2018). ShinyGO: a graphical enrichment tool for animals and plants. *bioRxiv*. 10.1093/bioinformatics/btz931 PMC7178415 31882993

[DMM045559C31] Gennebäck N., Hellman U., Malm L., Larsson G., Ronquist G., Waldenström A. and Mörner S. (2013). Growth factor stimulation of cardiomyocytes induces changes in the transcriptional contents of secreted exosomes. *J. Extracell. Vesicles* 2. 10.3402/jev.v2i0.20167 PMC3760655 24009898

[DMM045559C32] Gonzalez D. R., Treuer A. V., Lamirault G., Mayo V., Cao Y., Dulce R. A. and Hare J. M. (2014). NADPH oxidase-2 inhibition restores contractility and intracellular calcium handling and reduces arrhythmogenicity in dystrophic cardiomyopathy. *Am. J. Physiol. Heart Circ. Physiol.* 307, H710-H721. 10.1152/ajpheart.00890.2013 25015966 PMC4187396

[DMM045559C33] Grad L. I., Yerbury J. J., Turner B. J., Guest W. C., Pokrishevsky E., O'Neill M. A., Yanai A., Silverman J. M., Zeineddine R., Corcoran L. et al. (2010). Intercellular propagated misfolding of wild-type Cu/Zn superoxide dismutase occurs via exosome-dependent and -independent mechanisms. *Proc. Natl. Acad. Sci. USA* 111, 3620-3625. 10.1073/pnas.1312245111 PMC3948312 24550511

[DMM045559C34] Guan X., Mack D. L., Moreno C. M., Strande J. L., Mathieu J., Shi Y., Markert C. D., Wang Z., Liu G., Lawlor M. W. et al. (2014). Dystrophin-deficient cardiomyocytes derived from human urine: New biologic reagents for drug discovery. *Stem Cell Res.* 12, 467 10.1016/j.scr.2013.12.004 24434629 PMC3966181

[DMM045559C35] Guo J., Jayaprakash P., Dan J., Wise P., Jang G.-B., Liang C., Chen M., Woodley D. T., Fabbri M. and Li W. (2017). PRAS40 Connects Microenvironmental Stress Signaling to Exosome-Mediated Secretion. *Mol. Cell. Biol.* 19, e00171-17 10.1128/MCB.00171-17 PMC5599722 28674187

[DMM045559C36] Gupta S. and Knowlton A. A. (2007). HSP60 trafficking in adult cardiac myocytes: role of the exosomal pathway. *Am. J. Physiol. Heart Circ. Physiol.* 292, H3052-H3056. 10.1152/ajpheart.01355.2006 17307989

[DMM045559C37] Halkein J., Tabruyn S. P., Ricke-Hoch M., Haghikia A., Nguyen N.-Q.-N., Scherr M., Castermans K., Malvaux L., Lambert V., Thiry M. et al. (2013). MicroRNA-146a is a therapeutic target and biomarker for peripartum cardiomyopathy. *J. Clin. Invest.* 123, 2143-2154. 10.1172/JCI64365 23619365 PMC3638905

[DMM045559C38] Höck J. and Meister G. (2008). The Argonaute protein family. *Genome Biol.* 9, 210 10.1186/gb-2008-9-2-210 18304383 PMC2374724

[DMM045559C39] Ibrahim A. G.-E., Cheng K. and Marbán E. (2014). Exosomes as critical agents of cardiac regeneration triggered by cell therapy. *Stem Cell Rep.* 2, 606-619. 10.1016/j.stemcr.2014.04.006 PMC4050492 24936449

[DMM045559C40] Jung C., Martins A. S., Niggli E. and Shirokova N. (2008). Dystrophic cardiomyopathy: amplification of cellular damage by Ca2+ signalling and reactive oxygen species-generating pathways. *Cardiovasc. Res.* 77, 766-773. 10.1093/cvr/cvm089 18056762

[DMM045559C41] Khairallah M., Khairallah R. J., Young M. E., Allen B. G., Gillis M. A., Danialou G., Deschepper C. F., Petrof B. J. and Des Rosiers C. (2008). Sildenafil and cardiomyocyte-specific cGMP signaling prevent cardiomyopathic changes associated with dystrophin deficiency. *Proc. Natl. Acad. Sci. USA* 105, 7028-7033. 10.1073/pnas.0710595105 18474859 PMC2383977

[DMM045559C42] Khouzami L., Bourin M.-C., Christov C., Damy T., Escoubet B., Caramelle P., Perier M., Wahbi K., Meune C., Pavoine C. et al. (2010). Delayed cardiomyopathy in dystrophin deficient mdx mice relies on intrinsic glutathione resource. *Am. J. Pathol.* 177, 1356-1364. 10.2353/ajpath.2010.090479 20696779 PMC2928968

[DMM045559C43] Kourembanas S. (2015). Exosomes: vehicles of intercellular signaling, biomarkers, and vectors of cell therapy. *Annu. Rev. Physiol.* 77, 13-27. 10.1146/annurev-physiol-021014-071641 25293529

[DMM045559C44] Kuwahara K., Saito Y., Kishimoto I., Miyamoto Y., Harada M., Ogawa E., Hamanaka I., Kajiyama N., Takahashi N., Izumi T. et al. (2000). Cardiotrophin-1 phosphorylates akt and BAD, and prolongs cell survival via a PI3K-dependent pathway in cardiac myocytes. *J. Mol. Cell. Cardiol.* 32, 1385-1394. 10.1006/jmcc.2000.1177 10900165

[DMM045559C45] Lai R. C., Arslan F., Lee M. M., Sze N. S. K., Choo A., Chen T. S., Salto-Tellez M., Timmers L., Lee C. N., El Oakley R. M. et al. (2010). Exosome secreted by MSC reduces myocardial ischemia/reperfusion injury. *Stem Cell Res.* 4, 214-222. 10.1016/j.scr.2009.12.003 20138817

[DMM045559C46] Lauschke V. M., Vorrink S. U., Moro S. M. L., Rezayee F., Nordling Å., Hendriks D. F. G., Bell C. C., Sison-Young R., Park B. K., Goldring C. E. et al. (2016). Massive rearrangements of cellular MicroRNA signatures are key drivers of hepatocyte dedifferentiation. *Hepatology* 64, 1743-1756. 10.1002/hep.28780 27532775

[DMM045559C47] Lee C., Mitsialis S. A., Aslam M., Vitali S. H., Vergadi E., Konstantinou G., Sdrimas K., Fernandez-Gonzalez A. and Kourembanas S. (2012). Exosomes mediate the cytoprotective action of mesenchymal stromal cells on hypoxia-induced pulmonary hypertension. *Circulation* 126, 2601-2611. 10.1161/CIRCULATIONAHA.112.114173 23114789 PMC3979353

[DMM045559C48] Lee W.-H., Tsai M.-J., Chang W.-A., Wu L.-Y., Wang H.-Y., Chang K.-F., Su H.-M. and Kuo P.-L. (2018). Deduction of novel genes potentially involved in hypoxic AC16 human cardiomyocytes using next-generation sequencing and bioinformatics approaches. *Int. J. Mol. Med.* 42, 2489-2502. 10.3892/ijmm.2018.3851 30226555 PMC6192719

[DMM045559C49] Li Y., Zhang S., Zhang X., Li J., Ai X., Zhang L., Yu D., Ge S., Peng Y. and Chen X. (2014). Blunted cardiac beta-adrenergic response as an early indication of cardiac dysfunction in Duchenne muscular dystrophy. *Cardiovasc. Res.* 103, 60-71. 10.1093/cvr/cvu119 24812281 PMC4133593

[DMM045559C50] Li H., Liao Y., Gao L., Zhuang T., Huang Z., Zhu H. and Ge J. (2018). Coronary serum exosomes derived from patients with myocardial ischemia regulate angiogenesis through the miR-939-mediated nitric oxide signaling pathway. *Theranostics* 8, 2079-2093. 10.7150/thno.21895 29721064 PMC5928872

[DMM045559C51] Liao L., Zhou Q., Song Y., Wu W., Yu H., Wang S., Chen Y., Ye M. and Lu L. (2013). Ceramide mediates Ox-LDL-induced human vascular smooth muscle cell calcification via p38 mitogen-activated protein kinase signaling. *PLoS ONE* 12, e82379 10.1371/journal.pone.0082379 PMC3865066 24358176

[DMM045559C52] Liao Y., Smyth G. K. and Shi W. (2014). FeatureCounts: an efficient general purpose program for assigning sequence reads to genomic features. *Bioinformatics* 30, 923-930. 10.1093/bioinformatics/btt656 24227677

[DMM045559C53] Lijnen P. J., Petrov V. V. and Fagard R. H. (2000). Induction of cardiac fibrosis by transforming growth factor-β1. *Mol. Genet. Metab.* 71, 418-435. 10.1006/mgme.2000.3032 11001836

[DMM045559C54] Lin B., Li Y., Han L., Kaplan A. D., Ao Y., Kalra S., Bett G. C. L., Rasmusson R. L., Denning C. and Yang L. (2015). Modeling and study of the mechanism of dilated cardiomyopathy using induced pluripotent stem cells derived from individuals with Duchenne muscular dystrophy. *Dis. Model. Mech.* 8, 457 10.1242/dmm.019505 25791035 PMC4415895

[DMM045559C55] Lyu L., Wang H., Li B., Qin Q., Qi L., Nagarkatti M., Nagarkatti P., Janicki J. S., Wang X. L. and Cui T. (2015). A critical role of cardiac fibroblast-derived exosomes in activating renin angiotensin system in cardiomyocytes. *J. Mol. Cell. Cardiol.* 89, 268-279. 10.1016/j.yjmcc.2015.10.022 26497614 PMC4988239

[DMM045559C56] Madsen C., Hooper I., Lundberg L., Shafagati N., Johnson A., Senina S., De La Fuente C., Hoover L. I., Fredricksen B. L., Dinman J. et al. (2014). Small molecule inhibitors of Ago2 decrease Venezuelan equine encephalitis virus replication. *Antivir. Res.* 112, 26-37. 10.1016/j.antiviral.2014.10.002 25448087

[DMM045559C57] Mah J. K. (2016). Current and emerging treatment strategies for Duchenne muscular dystrophy. *Neuropsychiatr. Dis. Treat* 12, 1795-1807. 10.2147/NDT.S93873 27524897 PMC4966503

[DMM045559C58] Marques F. Z., Vizi D., Khammy O., Mariani J. A. and Kaye D. M. (2016). The transcardiac gradient of cardio-microRNAs in the failing heart. *Eur. J. Heart Fail* 18, 1000-1008. 10.1002/ejhf.517 27072074

[DMM045559C59] Menck K., Sönmezer C., Worst T. S., Schulz M., Dihazi G. H., Streit F., Erdmann G., Kling S., Boutros M., Binder C. et al. (2017). Neutral sphingomyelinases control extracellular vesicles budding from the plasma membrane. *J. Extracell. Vesicles* 6, 1378056 10.1080/20013078.2017.1378056 29184623 PMC5699186

[DMM045559C60] Mourkioti F., Kustan J., Kraft P., Day J. W., Zhao M.-M., Kost-Alimova M., Protopopov A., Depinho R. A., Bernstein D., Meeker A. K. et al. (2013). Role of telomere dysfunction in cardiac failure in Duchenne muscular dystrophy. *Nat. Cell Biol.* 15, 895-904. 10.1038/ncb2790 23831727 PMC3774175

[DMM045559C61] Németh A., Orgovan N., Sódar B. W., Osteikoetxea X., Pálóczi K., Szabó-Taylor K. É., Vukman K. V., Kittel Á., Turiák L., Wiener Z. et al. (2017). Antibiotic-induced release of small extracellular vesicles (exosomes) with surface-associated DNA. *Sci. Rep.* 7, 8202 10.1038/s41598-017-08392-1 28811610 PMC5557920

[DMM045559C62] Ovchinnikov D. A., Hidalgo A., Yang S.-K., Zhang X., Hudson J., Mazzone S. B., Chen C., Cooper-White J. J. and Wolvetang E. J. (2015). Isolation of contractile cardiomyocytes from human pluripotent stem-cell-derived cardiomyogenic cultures using a human NCX1-EGFP reporter. *Stem Cells Dev.* 24, 11-20. 10.1089/scd.2014.0195 25075536

[DMM045559C63] Pap E., Pállinger E., Pásztói M. and Falus A. (2009). Highlights of a new type of intercellular communication: microvesicle-based information transfer. *Inflamm. Res.* 58, 1-8. 10.1007/s00011-008-8210-7 19132498

[DMM045559C64] Pavoine C. and Pecker F. (2009). Sphingomyelinases: their regulation and roles in cardiovascular pathophysiology. *Cardiovasc. Res.* 82, 175-183. 10.1093/cvr/cvp030 19176603 PMC2855341

[DMM045559C65] Peter A. K. and Crosbie R. H. (2006). Hypertrophic response of Duchenne and limb-girdle muscular dystrophies is associated with activation of Akt pathway. *Exp. Cell Res.* 312, 2580-2591. 10.1016/j.yexcr.2006.04.024 16797529

[DMM045559C66] Quinlan J. G., Hahn H. S., Wong B. L., Lorenz J. N., Wenisch A. S. and Levin L. S. (2004). Evolution of the mdx mouse cardiomyopathy: physiological and morphological findings. *Neuromuscul. Disord.* 14, 491-496. 10.1016/j.nmd.2004.04.007 15336690

[DMM045559C67] Rajendran L., Honsho M., Zahn T. R., Keller P., Geiger K. D., Verkade P. and Simons K. (2006). Alzheimer's disease beta-amyloid peptides are released in association with exosomes. *Proc. Natl. Acad. Sci. USA* 30, 11172-11177. 10.1073/pnas.0603838103 PMC1544060 16837572

[DMM045559C68] Robinson M., Mccarthy D. and Smyth G. (2010). edgeR: a Bioconductor package for differential expression analysis of digital gene expression data. *Bioinformatics* 26, 139-140. 10.1093/bioinformatics/btp616 19910308 PMC2796818

[DMM045559C69] Rogers R. G., Fournier M., Sanchez L., Ibrahim A. G., Aminzadeh M. A., Lewis M. I. and Marbán E. (2019). Disease-modifying bioactivity of intravenous cardiosphere-derived cells and exosomes in mdx mice. *JCI Insight* 4, e125754 10.1172/jci.insight.125754 PMC6483717 30944252

[DMM045559C70] Schmidt M. F., Korb O. and Abell C. (2013). MicroRNA-specific argonaute 2 protein inhibitors. *ACS Chem. Biol.* 8, 2122-2126. 10.1021/cb400246k 23902134

[DMM045559C71] Shirokova N. and Niggli E. (2013). Cardiac phenotype of Duchenne muscular dystrophy: insights from cellular studies. *J. Mol. Cell. Cardiol.* 58, 217-224. 10.1016/j.yjmcc.2012.12.009 23261966 PMC3615054

[DMM045559C72] Singh R., Pochampally R., Watabe K., Lu Z. and Mo Y.-Y. (2014). Exosome-mediated transfer of miR-10b promotes cell invasion in breast cancer. *Mol. Cancer* 13, 256 10.1186/1476-4598-13-256 25428807 PMC4258287

[DMM045559C73] Spurney C. F., Knoblach S., Pistilli E. E., Nagaraju K., Martin G. R. and Hoffman E. P. (2008). Dystrophin-deficient cardiomyopathy in mouse: expression of Nox4 and Lox are associated with fibrosis and altered functional parameters in the heart. *Neuromuscul. Disord.* 18, 371-381. 10.1016/j.nmd.2008.03.008 18440230 PMC2430663

[DMM045559C74] Su X., Jin Y., Shen Y., Ju C., Cai J., Liu Y., Kim I., Wang Y., Yu H., Weintraub N. et al. (2018). Exosome-derived dystrophin from allograft myogenic progenitors improves cardiac function in Duchenne muscular dystrophic mice. *J. Cardiovasc. Transl. Res.* 11, 412-419. 10.1007/s12265-018-9826-9 30155598 PMC6212302

[DMM045559C75] Tabak S., Schreiber-Avissar S. and Beit-Yannai E. (2018). Extracellular vesicles have variable dose-dependent effects on cultured draining cells in the eye. *J. Cell. Mol. Med.* 22, 1992-2000. 10.1111/jcmm.13505 29411534 PMC5824413

[DMM045559C76] Tidball J. G. and Wehling-Henricks M. (2007). The role of free radicals in the pathophysiology of muscular dystrophy. *J. Appl. Physiol.* 102, 1677-1686. 10.1152/japplphysiol.01145.2006 17095633

[DMM045559C77] Trajkovic K., Hsu C., Chiantia S., Rajendran L., Wenzel D., Wieland F., Schwille P., Brugger B. and Simons M. (2008). Ceramide triggers budding of exosome vesicles into multivesicular endosomes. *Science* 319, 1244-1247. 10.1126/science.1153124 18309083

[DMM045559C78] Tyanova S., Temu T. and Cox J. (2016a). The MaxQuant computational platform for mass spectrometry-based shotgun proteomics. *Nat. Protoc.* 11, 2301-2319. 10.1038/nprot.2016.136 27809316

[DMM045559C79] Tyanova S., Temu T., Sinitcyn P., Carlson A., Hein M. Y., Geiger T., Mann M. and Cox J. (2016b). The Perseus computational platform for comprehensive analysis of (prote)omics data. *Nat. Methods* 13, 731-740. 10.1038/nmeth.3901 27348712

[DMM045559C80] Vicencio J. M., Yellon D. M., Sivaraman V., Das D., Boi-Doku C., Arjun S., Zheng Y., Riquelme J. A., Kearney J., Sharma V. et al. (2015). Plasma exosomes protect the myocardium from ischemia-reperfusion injury. *J. Am. Coll. Cardiol.* 65, 1525-1536. 10.1016/j.jacc.2015.02.026 25881934

[DMM045559C93] Vlachos I. S., Kostoulas N., Vergoulis T., Georgakilas G., Reczko M., Maragkakis M., Paraskevopoulou M. D., Prionidis K., Dalamagas T. and Hatzigeorgiou A. G. (2012). DIANA miRPath v.2.0: investigating the combinatorial effect of microRNAs in pathways. *Nucleic Acids Res.* 40, W498-W504. 10.1093/nar/gks494 22649059 PMC3394305

[DMM045559C81] Waldenström A., Gennebäck N., Hellman U. and Ronquist G. (2012). Cardiomyocyte microvesicles contain DNA/RNA and convey biological messages to target cells. *PLoS ONE* 7, e34653 10.1371/journal.pone.0034653 22506041 PMC3323564

[DMM045559C82] Wang X., Huang W., Liu G., Cai W., Millard R., Wang Y., Chang J., Peng T. and Fan G.-C. (2014a). Cardiomyocytes mediate anti-angiogenesis in type 2 diabetic rats through the exosomal transfer of miR-320 into endothelial cells. *J. Mol. Cell. Cardiol.* 74, 139-150. 10.1016/j.yjmcc.2014.05.001 24825548 PMC4120246

[DMM045559C83] Wang Y., Zhang L., Li Y., Chen L., Wang X., Guo W., Zhang X., Qin G., He S.-H., Zimmerman A. et al. (2015). Exosomes/microvesicles from induced pluripotent stem cells deliver cardioprotective miRNAs and prevent cardiomyocyte apoptosis in the ischemic myocardium. *Int. J. Cardiol.* 192, 61-69. 10.1016/j.ijcard.2015.05.020 26000464 PMC4469495

[DMM045559C84] Wang K., Jiang Z., Webster K. A., Chen J., Hu H., Zhou Y., Zhao J., Wang L., Wang Y., Zhong Z. et al. (2017). Enhanced cardioprotection by human endometrium mesenchymal stem cells driven by exosomal MicroRNA-21. *Stem Cells Transl. Med.* 6, 209-222. 10.5966/sctm.2015-0386 28170197 PMC5442741

[DMM045559C85] Webber J., Steadman R., Mason M., Tabi Z. and Clayton A. (2010). Cancer exosomes trigger fibroblast to myofibroblast differentiation. *Cancer Res.* 70, 9621-9630. 10.1158/0008-5472.CAN-10-1722 21098712

[DMM045559C86] Wehling-Henricks M., Jordan M. C., Gotoh T., Grody W. W., Roos K. P. and Tidball J. G. (2010). Arginine metabolism by macrophages promotes cardiac and muscle fibrosis in mdx muscular dystrophy. *PLoS ONE* 5, e10763 10.1371/journal.pone.0010763 20505827 PMC2874011

[DMM045559C87] Wu K., Hu M., Chen Z., Xiang F., Chen G., Yan W., Peng Q. and Chen X. (2017). Asiatic acid enhances survival of human AC16 cardiomyocytes under hypoxia by upregulating miR-1290. *IUBMB Life* 69, 660-667. 10.1002/iub.1648 28686797

[DMM045559C88] Xi H., Shin W. S., Suzuki J.-I., Nakajima T., Kawada T., Uehara Y., Nakazawa M. and Toyo-Oka T. (2000). Dystrophin disruption might be related to myocardial cell apoptosis caused by isoproterenol. *J. Cardiovasc. Pharmacol.* 36, S25-S29. 10.1097/00005344-200000006-00007 11206716

[DMM045559C89] Yu J., Novgorodov S. A., Chudakova D., Zhu H., Bielawska A., Bielawski J., Obeid L. M., Kindy M. S. and Gudz T. I. (2007). JNK3 signaling pathway activates ceramide synthase leading to mitochondrial dysfunction. *J. Biol. Chem.* 282, 25940-25949. 10.1074/jbc.M701812200 17609208

[DMM045559C90] Yu B., Kim H. W., Gong M., Wang J., Millard R. W., Wang Y., Ashraf M. and Xu M. (2015). Exosomes secreted from GATA-4 overexpressing mesenchymal stem cells serve as a reservoir of anti-apoptotic microRNAs for cardioprotection. *Int. J. Cardiol.* 182, 349-360. 10.1016/j.ijcard.2014.12.043 25590961 PMC4382384

[DMM045559C91] Zanotti S., Gibertini S., Blasevich F., Bragato C., Ruggieri A., Saredi S., Fabbri M., Bernasconi P., Maggi L., Mantegazza R. et al. (2018). Exosomes and exosomal miRNAs from muscle-derived fibroblasts promote skeletal muscle fibrosis. *Matrix Biol.* 74, 77-100. 10.1016/j.matbio.2018.07.003 29981373

[DMM045559C92] Zhang J., Ma J., Long K., Qiu W., Wang Y., Hu Z., Liu C., Luo Y., Jiang A., Jin L. et al. (2017). Overexpression of exosomal cardioprotective miRNAs mitigates hypoxia-induced H9c2 cells apoptosis. *Int. J. Mol. Sci.* 18, 711 10.3390/ijms18040711 PMC5412297 28350318

