# Supplementary material for: Duchenne muscular dystrophy (DMD) cardiomyocyte-secreted exosomes promote the pathogenesis of DMD-associated cardiomyopathy
Source: Dis Model Mech. 2020 Nov 13;13(11):dmm045559. doi: 10.1242/dmm.045559 (PMC7673361; doi:10.1242/dmm.045559)
Supplement: Supplementary information [file dmm-13-045559-s1.pdf]

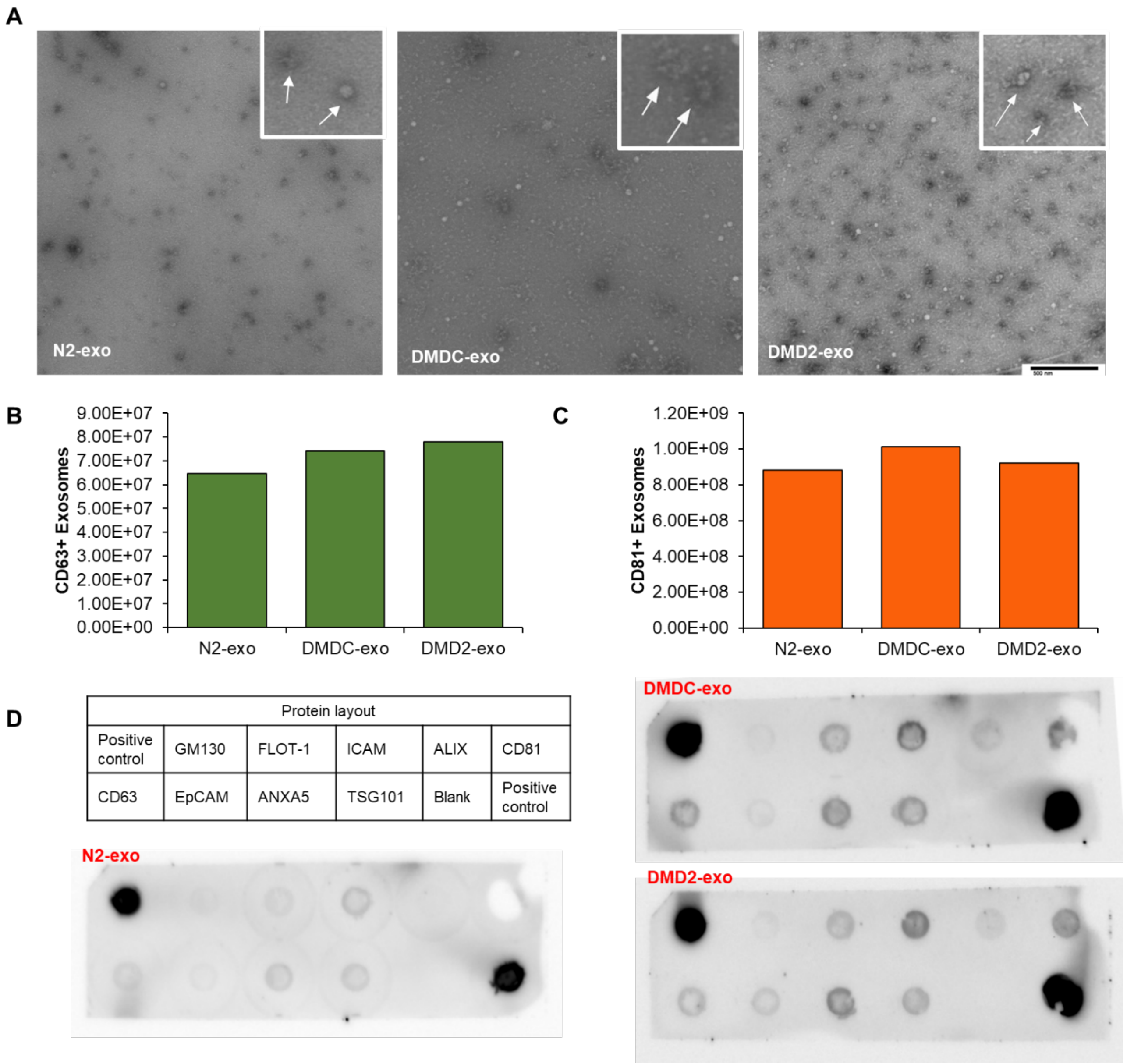

Figure S1. Characterization of exosomes. A) Exosomes were isolated from N2-, DMDC- and DMD2-iCMs and evaluated by electron microscopy. Exosomes displayed traditional exosome morphology and were ~40-50nm. Isolated exosomes were confirmed to express **B)** CD63 and **C)** CD81 by ELISA. Data represents mean exosome concentration of biological triplicates pooled across duplicate wells. N=3 biological replicates/ group. **D)** Dot blot array analysis showed that isolated exosomes display typical exosomal surface markers. Data represents pooled biological triplicates across a single membrane for each group. N=3 biological replicate exosome preparations pooled on a single membrane.

Disease Models & Mechanisms • Supplementary information

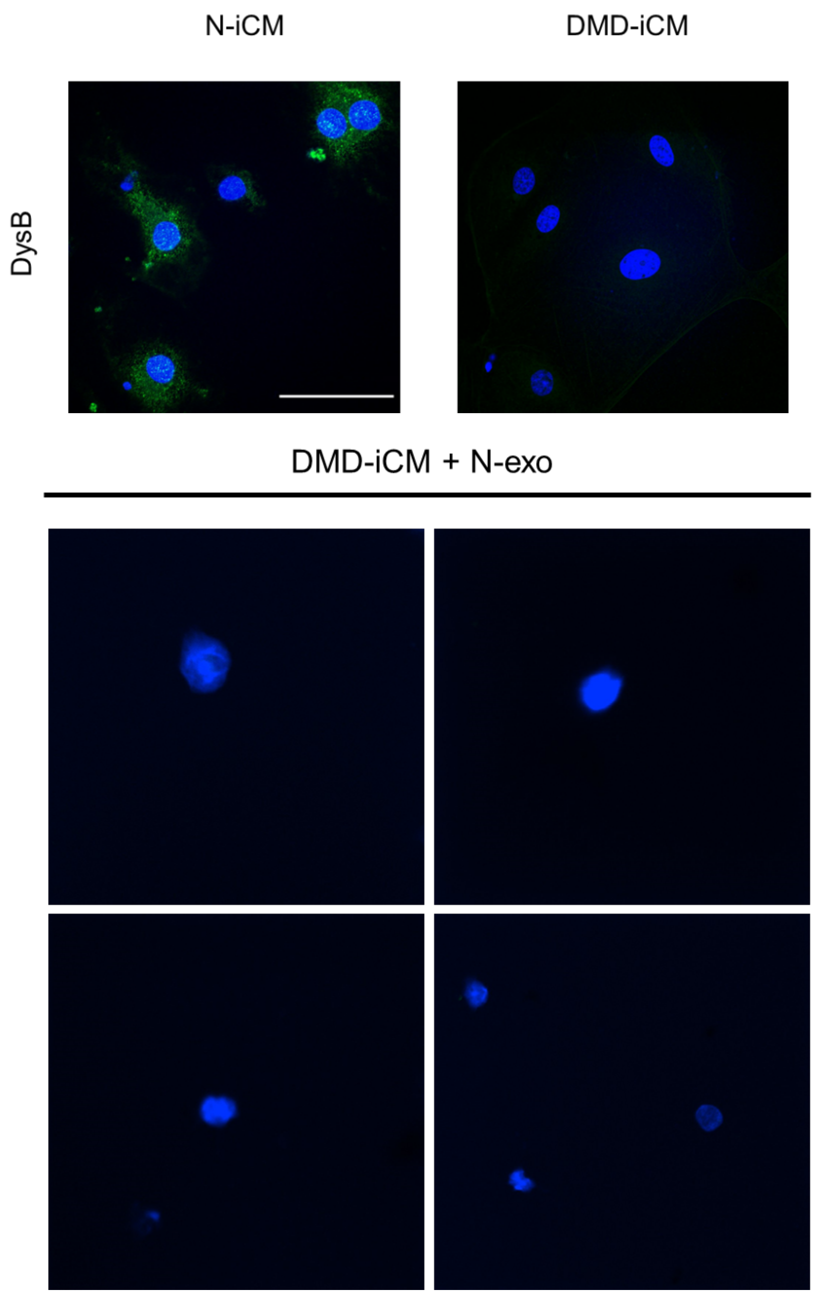

Figure S2. Exposure to N-exo for 48 hr does not result in dystrophin expression in DMD-iCMs. Cardiomyocytes were fixed and stained with NCL-DysB to evaluate dystrophin levels. Images were taken by laser scanning confocal microscopy and represent images taken at many different fields of depth and flattened into a single image. DMD-iCMs exposed to 48hr N-exo do not display dystrophin expression as shown by immunofluorescent staining. N=3 coverslips, 10 image fields per coverslip were imaged.

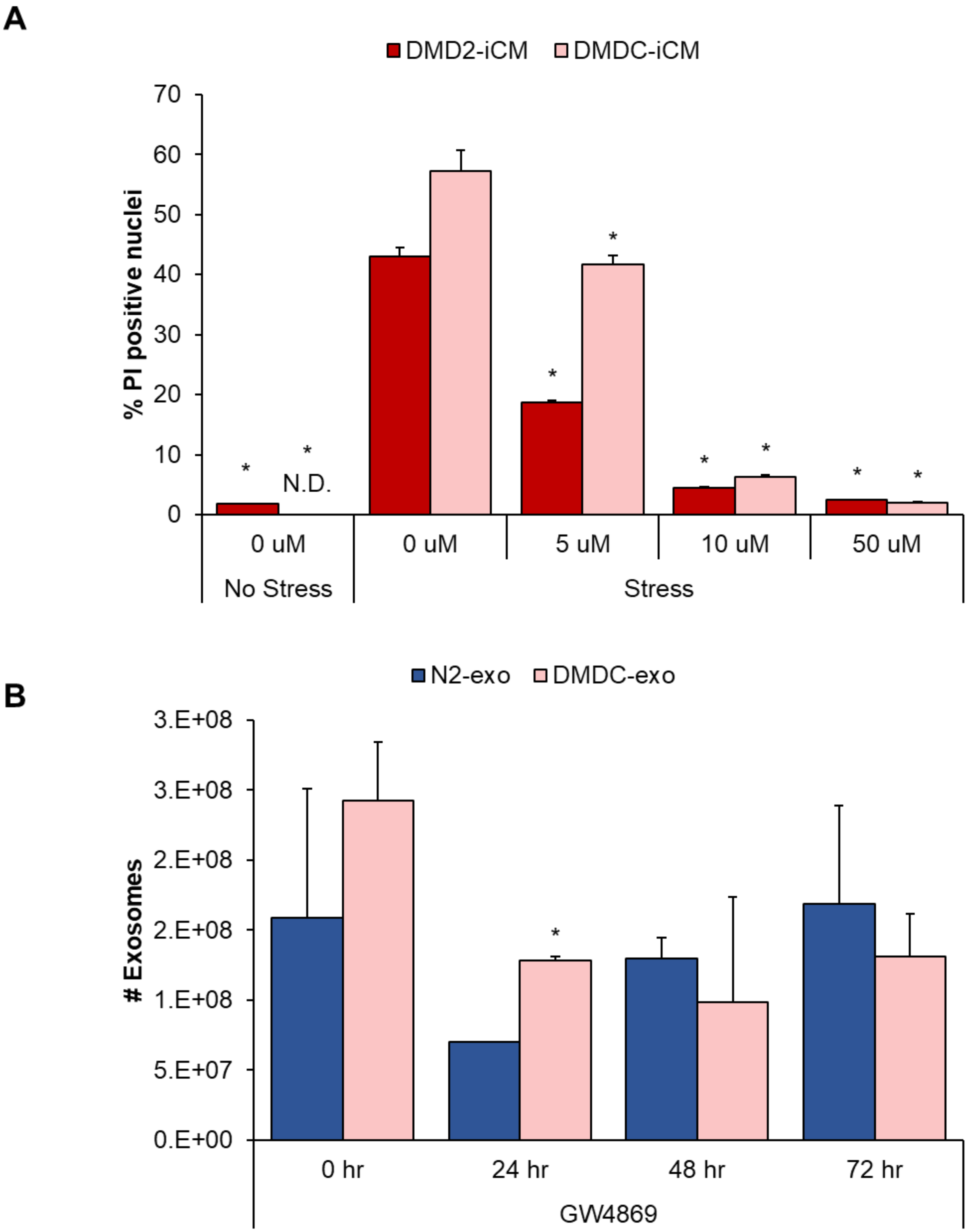

Figure S3. Optimizing the use of GW4869 to inhibit exosome release *in vitro*. A) Testing various concentrations of GW4869 in DMD-iCMs shows that inhibiting DMD-exo release significantly reduces stress-induced cell death starting at 10 uM, which was used for subsequent experiments. N=109-612 cells counted in each group; *p<0.05 vs. Stress + 0 uM. **B)** Exosome quantitation assays reveal that GW4869 significantly reduced exosome release 24hr after treatment. Data represent mean ± SEM. Significance was determined using a Student's T-test. N=3 biological replicates/group; p<0.05 GW4869 vs. 0 hr.

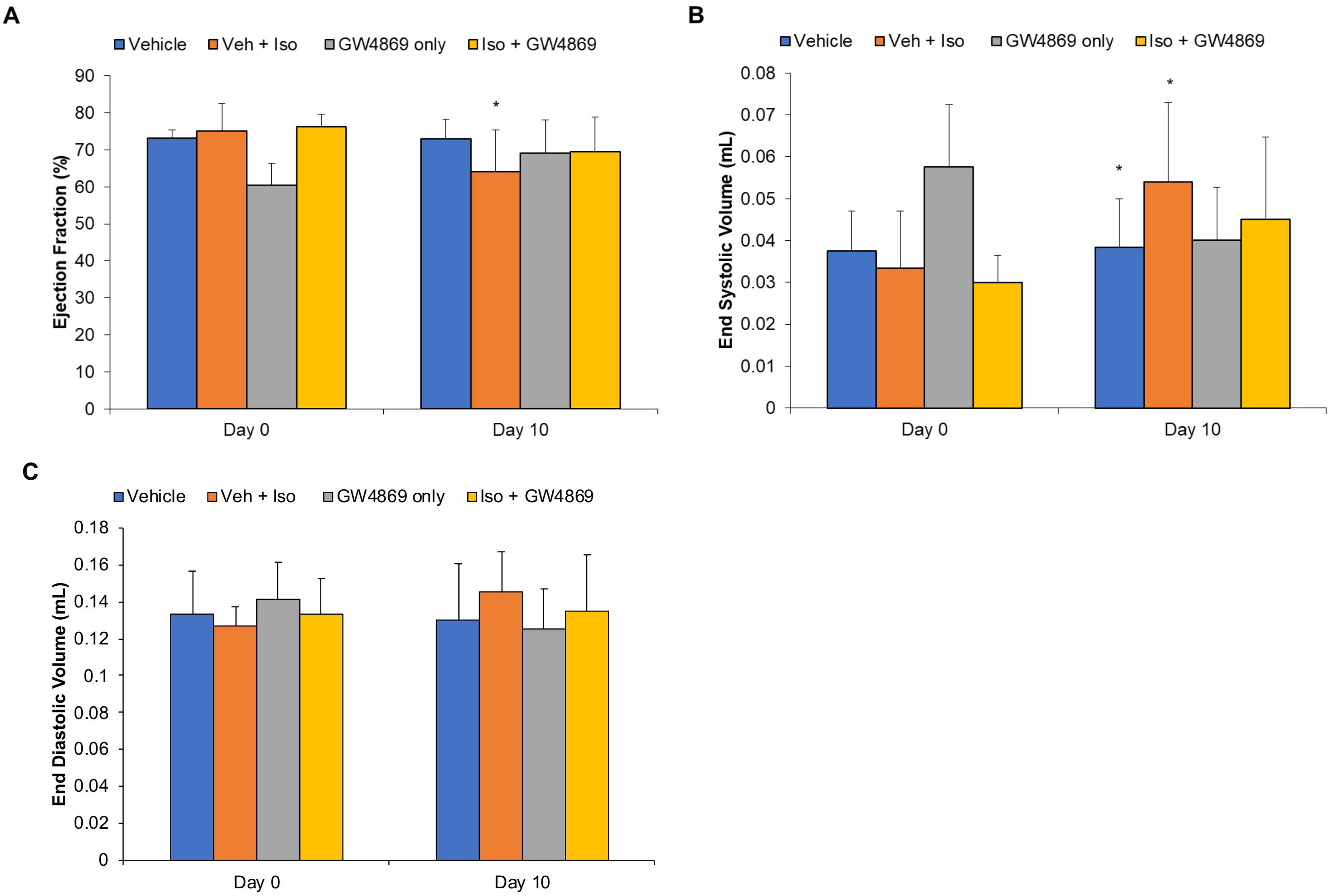

Figure S4. Exosome inhibition with GW4869 does not alter cardiac function in *mdx* mice. Following 10 days of exosome inhibition with GW4869 and isoproterenol stress *in vivo*, cardiac function in *mdx* mice was assess by echocardiography. **A)** Isoproterenol stress led to a decrease in % ejection fraction, **B)** and an increase in end systolic volume, but no change in **C)** end diastolic volume. No significant changes in cardiac function were seen with exosome inhibition. Data represent mean ± SEM. Significance was determined using a one-way ANOVA. N=6 animals/group; *$p<0.05$ vs. Day 0 under same conditions.

Disease Models & Mechanisms • Supplementary information

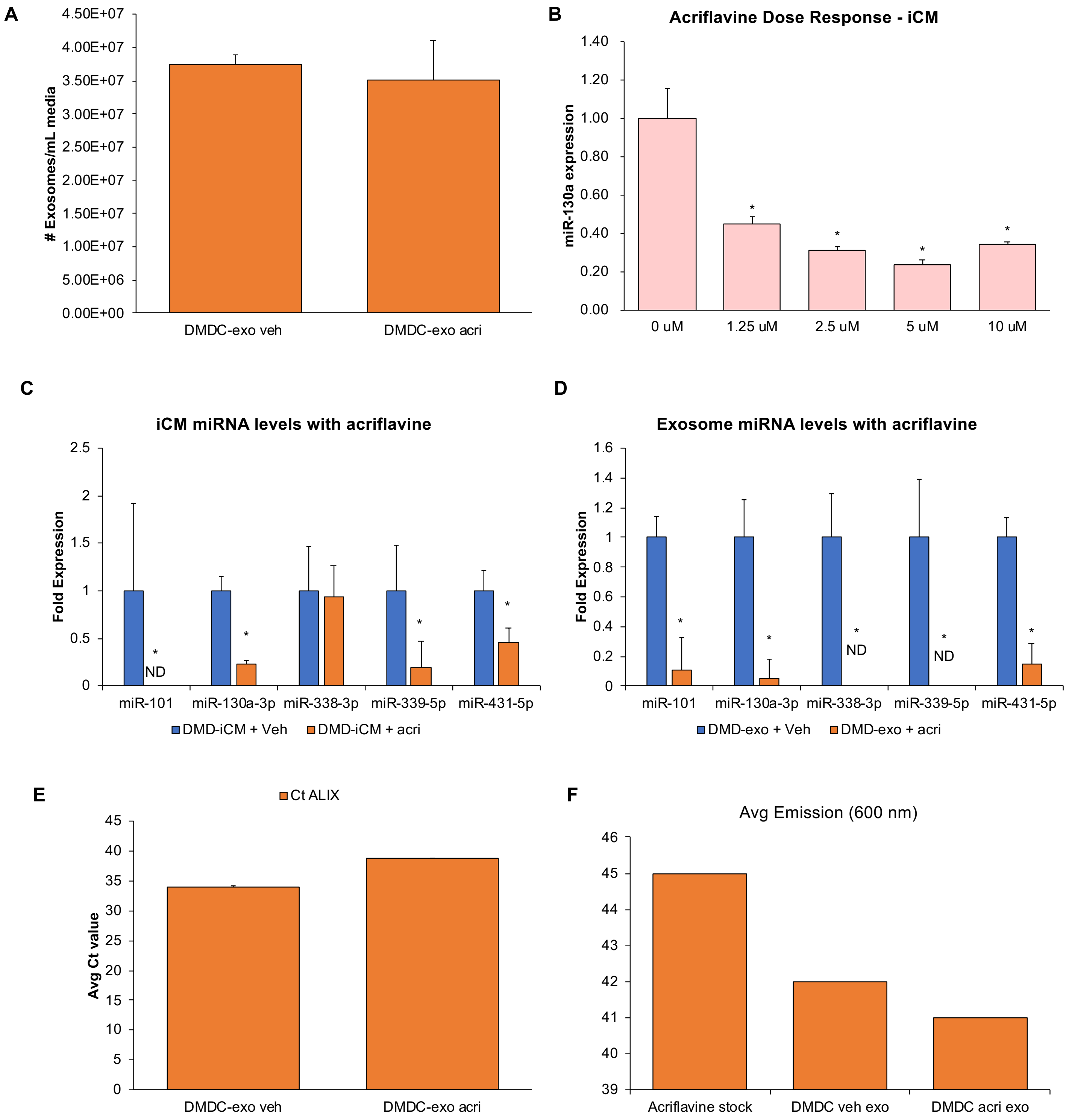

Figure S5. Optimizing the use of acriflavine *in vitro*. To confirm whether acriflavine had off-target effects on exosome production, exosomes were harvested to assess quantity. **A)** Exosome quantity is not significantly altered with acriflavine treatment as shown by exosome quantitation assay. N=3/group. **B)** Various concentrations of acriflavine were tested in DMD-iCM, followed by qPCR assessment of miR-130a expression (normalized to U6) with 5 uM determined to be the optimal dose to knockdown miR expression. N=3/group. **C)** qPCR of cardiomyocyte miRNA levels reveals downregulation of miR-130a-3p, miR-339-5p and miR-431-5p with acriflavine exposure. **D)** qPCR of cardiac exosome miRNA levels reveals downregulation of miR-101, miR-130a-3p, miR-338-3p, miR-339-5p and miR-431-5p following acriflavine exposure. **E)** Exosome mRNA levels are not altered by acriflavine treatment, as shown by qPCR for exosomal gene ALIX. Data represent mean ± SEM. Significance was determined using a Student's T-test. N=3 biological replicates/group.
F) Acriflavine is a fluorescent compound. To detect whether acriflavine was present in exosomes and may therefore exert biologic effects in cells treated with acriflavine miR-depleted exosomes, fluorescence was

examined as indication of the presence of the compound. Fluorescent absorbance was read on a plate reader at 600 nm which showed reduced fluorescence of DMD vehicle or acriflavine exosomes in comparison to the acriflavine stock, indicating it was not present in exosomes.

Table S1: Exosomal surface peptides differentially expressed on DMD- vs. N-exo

DMD-exosomes		N-exosomes	
**Protein Description**	**Gene Name**	**Protein Description**	**Gene Name**
Septin-9	SEPT9	Inactive serine protease 35	PRSS35
PDZ and LIM domain protein 7	PDLIM7	Collagen alpha-1 (XVI) chain	COL16A1
Reticulon-4	RTN4	Brain acid soluble protein 1	BASP1
Drebrin	DBN1	Myosin-6	MYH6
LIM and SH3 domain protein 1	LASP1	Proenkephalin-A<br>Synenkephalin<br>Met-enkephalin<br>PENK (114-133); PENK (143-183)<br>Met-enkephalin-Arg-Gly-Leu;Leu-enkephalin<br>PENK (237-258)<br>Met-enkephalin-Arg-Phe	PENK
Dynactin subunit 1	DCTN1	Collagen alpha-1 (I) chain	COL1A1
Transcription intermediary factor 1-beta	TRIM28	Histone H1.4;<br>Histone H1.1	HIST1H1E<br>HIST1H1A
14-3-3 protein gamma<br>14-3-3 protein gamma, N-terminally processed<br>14-3-3 protein beta/alpha<br>14-3-3 protein beta/alpha, N-terminally processed	YWHAG<br>YWHAB	Collagen alpha-2 (I) chain	COL1A2
Microtubule-associated protein 4	MAP4	Collagen alpha-3 (VI) chain	COL6A3
Heat shock protein HSP 90-beta	HSP90AB1	Histone H2B type 2-E<br>Histone H2B type 1-C/E/F/G/I<br>Histone H2B type 1-L<br>Histone H2B type 1-M<br>Histone H2B type 1-N<br>Histone H2B type 1-D<br>Histone H2B type 1-B<br>Histone H2B type 1-A<br>Histone H2B type 3-B	HIST2H2BE<br>HIST1H2BC<br>HIST1H2BL<br>HIST1H2B<br>M<br>HIST1H2BN<br>HIST1H2BD<br>HIST1H2BB<br>HIST1H2BA<br>HIST3H2BB
Glia-derived nexin	SERPINE2	Collagen alpha-1 (III) chain	COL3A1
Tropomyosin alpha-1 chain	TPM1	Myosin-7	MYH7
Pyruvate kinase PKM	PKM	Histone H4	HIST1H4A
Tubulin beta chain<br>Tubulin beta-2B chain<br>Tubulin beta-2A chain<br>Tubulin beta-3 chain	TUBB<br>TUBB2B<br>TUBB2A<br>TUBB3	Latent-transforming growth factor beta-binding protein 2	LTBP2
Glyceraldehyde-3-phosphate dehydrogenase	GAPDH	Secretogranin-2<br>Secretoneurin<br>Manserin	SCG2
Cytoplasmic dynein 1 heavy chain 1	DYNC1H1	60S ribosomal protein L29	RPL29
Actin, gamma-enteric smooth muscle<br>Actin, alpha cardiac muscle 1<br>Actin, aortic smooth muscle<br>Actin, alpha skeletal muscle	ACTG2<br>ACTC1<br>ACTA2<br>ACTA1	Histone H1.0<br>Histone H1.0, N-terminally processed	H1F0
Filamin-A	FLNA	Collagen alpha-1 (XI) chain	COL11A1

Dihydropyrimidinase-related protein 3	DPYSL3	Histone H3.2<br>Histone H3.1t<br>Histone H3.1<br>Histone H3.3C<br>Histone H3.3	HIST2H3A<br>HIST3H3<br>HIST1H3A<br>H3F3C<br>H3F3A
Actin, cytoplasmic 2<br>Actin, cytoplasmic 2, N-terminally processed<br>Actin, cytoplasmic 1<br>Actin, cytoplasmic 1, N-terminally processed	ACTG1<br>ACTB	Protein-lysine 6-oxidase	LOX
Filamin-B	FLNB	Tenascin	TNC
Transitional endoplasmic reticulum ATPase	VCP	Apolipoprotein C-III	APOC3
Tropomyosin alpha-4 chain	TPM4	Histone H2B type 1-H<br>Histone H2B type 2-F<br>Histone H2B type 1-O	HIST1H2BH<br>HIST2H2BF<br>HIST1H2BO
Heat shock cognate 71 kDa protein	HSPA8	Collagen alpha-2 (V) chain	COL5A2
Plectin	PLEC	60S ribosomal protein L7	RPL7
Septin-11	SEPT11		
Ribosome-binding protein 1	RRBP1		
Caldesmon	CALD1		
Heat shock protein beta-1	HSPB1		
Keratin, type I cytoskeletal 18	KRT18		
Talin-1	TLN1		
Vimentin	VIM		
Protein transport protein Sec23A	SEC23A		
Alpha-enolase	ENO1		
Spectrin beta chain, non-erythrocytic 1	SPTBN1		
Septin-7	SEPT7		
Myosin-11	MYH11		
Fibrillin-2	FBN2		
Spectrin alpha chain, non-erythrocytic 1	SPTAN1		
60 kDa heat shock protein, mitochondrial	HSPD1		
Protein AMBP<br>Alpha-1-microglobulin<br>Inter-alpha-trypsin inhibitor light chain<br>Trypstatin	AMBP		
Zyxin	ZYX		
Clathrin heavy chain 1	CLTC		
Tubulin alpha-1C chain<br>Tubulin alpha-1A chain<br>Tubulin alpha-1B chain<br>Tubulin alpha-3E chain	TUBA1C<br>TUBA1A<br>TUBA1B<br>TUBA3E		
78 kDa glucose-regulated protein	HSPA5		
Annexin A2<br>Putative annexin A2-like protein	ANXA2<br>ANXA2P2		

Septin-2	SEPT2	
Prelamin-A/C<br>Lamin-A/C	LMNA	
Alpha-enolase	ENO1	
Septin-9	SEPT9	

Table S2: DMD-exo peptides GO Analysis – Gene list

DMD-exo peptides		
**GO Category**	**Process**	**Genes**
**Biological Process**	Cytoskeleton organization	MAP4 TPM1 VCP TPM4 PLEC DYNC1H1 KRT18 FLNA DCTN1 VIM DPYSL3 DBN1 SPTBN1 FLNB TLN1 LMNA PDLIM7 SPTAN1 CLTC ZYX GAPDH MYH11
	Organelle organization	MAP4 SEC23A SEPT7 TPM1 HSPD1 VCP TPM4 SEPT2 PLEC DYNC1H1 HSP90AB1 KRT18 RTN4 FLNA DCTN1 VIM DPYSL3 DBN1 SPTBN1 TRIM28 SERPINE2 FLNB TLN1 LMNA PDLIM7 SPTAN1 CLTC ZYX SEPT9 GAPDH MYH11
	Supramolecular fiber organization	TPM1 TPM4 DYNC1H1 HSPA8 FLNA DCTN1 VIM DPYSL3 DBN1 SPTBN1 SPTAN1 HSP90AB1 MYH11 ZYX
	Cellular component assembly	SEC23A SEPT7 CLTC SEPT2 PLEC HSP90AB1 RTN4 TRIM28 SEPT11 VCP SEPT9 FLNA DCTN1 PKM DPYSL3 SPTBN1 TLN1 DYNC1H1 SPTAN1 MAP4 MYH11 TPM1 ZYX HSPD1 HSPA8
	Response to unfolded protein	HSPA5 HSPA8 HSPD1 HSP90AB1 HSPB1 TLN1 LMNA VCP DCTN1
	Cellular component biogenesis	SEC23A SEPT7 CLTC SEPT2 PLEC HSP90AB1 RTN4 TRIM28 SEPT11 VCP SEPT9 FLNA DCTN1 PKM DPYSL3 SPTBN1 TLN1 DYNC1H1 SPTAN1 MAP4 MYH11 TPM1 ZYX HSPD1 HSPA8
	Regulation of cellular component organization	ENO1 HSPA8 RTN4 VCP DCTN1 VIM HSPA5 DPYSL3 DBN1 SPTBN1 TRIM28 SERPINE2 SEPT11 LMNA FLNA SPTAN1 MAP4 SEPT7 TPM1 CLTC SEPT9 DYNC1H1
	Response to topologically incorrect protein	HSPA5 HSPA8 VCP HSPD1 HSP90AB1 HSPB1 TLN1 LMNA DCTN1
	Plasma membrane bounded cell projection organization	MAP4 RTN4 SEPT7 SEPT2 VIM HSPA5 HSP90AB1 DPYSL3 DBN1 SEPT9 FLNA DCTN1 TPM1 SPTBN1 PDLIM7 DYNC1H1 SPTAN1
	Actin filament-based process	TPM1 TPM4 FLNA DPYSL3 DBN1 SPTBN1 FLNB TLN1 PDLIM7 SPTAN1 ZYX MYH11 VIM
**Cellular Component**	Cytoskeleton	SEPT7 SEPT11 TPM1 CLTC TPM4 SEPT2 PLEC SEPT9 DYNC1H1 FLNA VIM MAP4 KRT18 DBN1 CALD1 ZYX PDLIM7 SPTAN1 DCTN1 LASP1 HSPB1 GAPDH DPYSL3 SPTBN1 MYH11 FLNB TLN1 LMNA
	Cytoskeletal part	SEPT7 SEPT11 TPM1 CLTC TPM4 SEPT2 PLEC SEPT9 DYNC1H1 VIM MAP4 KRT18 ZYX FLNA DCTN1 LASP1 HSPB1 DPYSL3 SPTBN1 CALD1 MYH11 FLNB LMNA PDLIM7 DBN1 SPTAN1
	Actin cytoskeleton	TPM1 TPM4 FLNA SEPT7 CALD1 SEPT11 ZYX SEPT9 PDLIM7 LASP1 DPYSL3 DBN1 SPTBN1 MYH11 FLNB DCTN1 SPTAN1
	Cell cortex part	SEPT7 SEPT11 SEPT2 SEPT9 ENO1 DCTN1 LASP1 SPTBN1 CALD1 TPM4 FLNA DBN1 SPTAN1
	Cell cortex	SEPT7 SEPT11 SEPT2 SEPT9 ENO1 DCTN1 LASP1 DBN1 SPTBN1 CALD1 FLNB TPM4 FLNA SPTAN1
	Supramolecular complex	TPM1 TPM4 PLEC VIM KRT18 FBN2 CLTC SEPT9 FLNA DYNC1H1 DCTN1 MAP4 ENO1 HSPB1 DPYSL3 SPTBN1 CALD1 FLNB LMNA MYH11
	Supramolecular polymer	TPM1 TPM4 PLEC VIM KRT18 FBN2 CLTC SEPT9 FLNA DYNC1H1 DCTN1 MAP4 ENO1 HSPB1 DPYSL3 SPTBN1 CALD1 FLNB LMNA MYH11

Disease Models & Mechanisms • Supplementary information

	Supramolecular fiber	TPM1 TPM4 PLEC VIM KRT18 FBN2 CLTC SEPT9 FLNA DYNC1H1 DCTN1 MAP4 ENO1 HSPB1 DPYSL3 SPTBN1 CALD1 FLNB LMNA MYH11
	Cytoplasmic region	SEPT7 SEPT11 SEPT2 SEPT9 MAP4 ENO1 DCTN1 LASP1 HSPB1 DBN1 SPTBN1 CALD1 FLNB TPM4 FLNA SPTAN1
	Non-membrane-bounded organelle	SEPT7 TRIM28 SEPT11 TPM1 CLTC TPM4 SEPT2 PLEC SEPT9 DYNC1H1 FLNA VIM MAP4 KRT18 GAPDH DBN1 SPTBN1 CALD1 ZYX VCP PDLIM7 SPTAN1 DCTN1 LASP1 ENO1 HSPB1 HSPA8 DPYSL3 MYH11 FLNB TLN1 LMNA RRBP1
**Molecular Function**	Cadherin binding	LASP1 HSPA5 PKM ENO1 HSP90AB1 HSPA8 KRT18 DBN1 SPTBN1 RTN4 SEPT7 CALD1 FLNB TLN1 SEPT2 PLEC SEPT9 FLNA SPTAN1
	Cell adhesion molecule binding	LASP1 HSPA5 PKM ENO1 HSP90AB1 HSPA8 KRT18 DBN1 SPTBN1 RTN4 SEPT7 CALD1 FLNB TLN1 SEPT2 PLEC SEPT9 FLNA SPTAN1
	Structural molecule activity	SEPT7 SEPT11 FBN2 SEPT2 PLEC SEPT9 VIM KRT18 SPTBN1 TLN1 CLTC LMNA MYH11 MAP4 TPM1 TPM4 SPTAN1
	Cytoskeletal protein binding	MAP4 TPM1 TPM4 PLEC FLNA DCTN1 LASP1 HSP90AB1 DBN1 SPTBN1 CALD1 MYH11 FLNB TLN1 SPTAN1 DPYSL3 GAPDH
	RNA binding	MAP4 PKM ENO1 HSP90AB1 HSPB1 HSPA8 KRT18 SPTBN1 RTN4 RRBP1 TRIM28 FLNB CLTC HSPD1 ZYX VCP PLEC FLNA DYNC1H1 VIM
	Actin binding	TPM1 TPM4 FLNA LASP1 DBN1 SPTBN1 CALD1 MYH11 FLNB TLN1 PLEC SPTAN1
	Protein-containing complex binding	PKM HSP90AB1 HSPA8 TPM1 TPM4 AMBP HSPD1 FLNA LASP1 HSPA5 SPTBN1 MYH11 TLN1 VCP DCTN1 VIM
	Structural constituent of cytoskeleton	PLEC VIM SPTBN1 TLN1 TPM1 SPTAN1
	Nucleoside-triphosphatase activity	HSPA5 HSPA8 SEPT7 SEPT11 VCP SEPT2 SEPT9 DYNC1H1 MYH11 DCTN1 HSPD1 HSP90AB1
	Pyrophosphatase activity	HSPA5 HSPA8 SEPT7 SEPT11 VCP SEPT2 SEPT9 DYNC1H1 MYH11 DCTN1 HSPD1 HSP90AB1

Disease Models & Mechanisms • Supplementary information

Table S3: DMD-exo peptides KEGG Analysis – Gene list

DMD-exo peptides	
**KEGG Pathway**	**Genes**
Protein processing in endoplasmic reticulum	SEC23A HSPA5 HSPA8 HSP90AB1 RRBP1 VCP
Bacterial invasion of epithelial cells	SEPT9 CLTC SEPT2 SEPT11
Legionellosis	HSPA8 HSPD1 VCP
Glycolysis / Gluconeogenesis	ENO1 GAPDH PKM
Biosynthesis of amino acids	ENO1 GAPDH PKM
Focal adhesion	FLNA FLNB TLN1 ZYX
Antigen processing and presentation	HSPA5 HSPA8 HSP90AB1
Salmonella infection	DYNC1H1 FLNA FLNB
Hypertrophic cardiomyopathy (HCM)	LMNA TPM1 TPM4
Dilated cardiomyopathy (DCM)	LMNA TPM1 TPM4
Carbon metabolism	ENO1 GAPDH PKM
MAPK signaling pathway	FLNA FLNB HSPA8 HSPB1
Estrogen signaling pathway	HSPA8 HSP90AB1 KRT18
Vasopressin-regulated water reabsorption	DCTN1 DYNC1H1
RNA degradation	ENO1 HSPD1
Cardiac muscle contraction	TPM1 TPM4
HIF-1 signaling pathway	ENO1 GAPDH
Apoptosis	LMNA SPTAN1
Adrenergic signaling in cardiomyocytes	TPM1 TPM4
MicroRNAs in cancer	TPM1 VIM
Alzheimer disease	GAPDH RTN4

Disease Models & Mechanisms • Supplementary information

Table S4: N-exo peptides GO Analysis – Gene list

N-exo peptides		
**GO Category**	**Process**	**Genes**
**Biological Process**	Extracellular structure organization	COL11A1 COL16A1 COL1A1 COL1A2 COL3A1 COL5A2 APOC3 LOX TNC COL6A3
	Collagen fibril organization	COL5A2 COL11A1 LOX COL3A1 COL1A1 COL1A2
	Extracellular matrix organization	COL11A1 COL16A1 COL1A1 COL1A2 COL3A1 COL5A2 LOX TNC COL6A3
	Cellular response to amino acid stimulus	COL16A1 COL1A1 COL1A2 COL3A1 COL5A2
	Animal organ morphogenesis	COL6A3 TNC COL11A1 COL1A1 COL3A1 MYH7 COL1A2 MYH6 COL5A2 BASP1
	Cellular response to acid chemical	TNC COL16A1 COL1A1 COL1A2 COL3A1 COL5A2
	Ossification	TNC COL1A1 COL11A1 LOX COL1A2 PENK COL5A2
	Muscle organ development	COL11A1 LOX COL3A1 MYH6 MYH7 BASP1 COL6A3
	Response to amino acid	COL16A1 COL1A1 COL1A2 COL3A1 COL5A2
	Cellular response to transforming growth factor beta stimulus	COL1A2 COL3A1 COL1A1 LOX PENK LTBP2
**Cellular Component**	Collagen trimer	COL11A1 COL1A1 COL1A2 COL3A1 COL5A2 COL16A1 LOX COL6A3
	Endoplasmic reticulum lumen	TNC COL11A1 COL16A1 COL1A1 COL6A3 COL1A2 COL3A1 SCG2 PENK COL5A2
	Extracellular matrix	TNC COL11A1 COL16A1 COL1A1 APOC3 LTBP2 COL6A3 COL1A2 COL3A1 COL5A2 LOX
	Fibrillar collagen trimer	COL11A1 COL1A1 COL1A2 COL3A1 COL5A2
	Collagen-containing extracellular matrix	TNC COL11A1 COL16A1 COL1A1 APOC3 LTBP2 COL6A3 COL1A2 COL3A1 COL5A2
	Banded collagen fibril	COL11A1 COL1A1 COL1A2 COL3A1 COL5A2
	Extracellular matrix component	COL11A1 COL1A1 COL1A2 COL3A1 COL5A2 TNC
	Complex of collagen trimers	COL11A1 COL1A1 COL1A2 COL3A1 COL5A2
	Extracellular region	TNC COL11A1 COL16A1 COL1A1 APOC3 LTBP2 COL6A3 COL1A2 COL3A1 BASP1 COL5A2 HIST1H4A LOX SCG2 PRSS35 PENK
	Extracellular space	TNC COL1A1 APOC3 LTBP2 COL6A3 COL1A2 BASP1 HIST1H4A COL11A1 COL16A1 LOX COL3A1 SCG2 COL5A2
**Molecular Function**	Extracellular matrix structural constituent conferring tensile strength	COL11A1 COL16A1 COL1A1 COL6A3 COL1A2 COL3A1 COL5A2
	Extracellular matrix structural constituent	COL11A1 COL16A1 COL1A1 COL1A2 COL3A1 COL5A2 TNC LTBP2 COL6A3

Disease Models & Mechanisms • Supplementary information

	Structural molecule activity	COL11A1 COL16A1 COL1A1 RPL7 RPL29 COL1A2 COL3A1 COL5A2 TNC LTBP2 COL6A3
	Platelet-derived growth factor binding	COL1A1 COL1A2 COL3A1
	Growth factor binding	COL1A1 COL1A2 COL3A1 LTBP2
	Actin-dependent ATPase activity	MYH7 MYH6
	SMAD binding	COL1A2 COL3A1 COL5A2
	Microfilament motor activity	MYH7 MYH6
	Protease binding	COL1A1 COL1A2 COL3A1
	Heparin binding	COL11A1 LTBP2 RPL29

Disease Models & Mechanisms • Supplementary information

Table S5: N-exo peptides KEGG Analysis – Gene list

N-exo peptides	
**KEGG Pathway**	**Genes**
Protein digestion and absorption	COL1A1 COL1A2 COL3A1 COL5A2 COL6A3 COL11A1
ECM-receptor interaction	COL1A1 COL1A2 COL6A3 TNC
Focal adhesion	COL1A1 COL1A2 COL6A3 TNC
AGE-RAGE signaling pathway in diabetic complications	COL1A1 COL1A2 COL3A1
Amoebiasis	COL1A1 COL1A2 COL3A1
Platelet activation	COL1A1 COL1A2 COL3A1
Relaxin signaling pathway	COL1A1 COL1A2 COL3A1
Human papillomavirus infection	COL1A1 COL1A2 COL6A3 TNC
PI3K-Akt signaling pathway	COL1A1 COL1A2 COL6A3 TNC
Viral myocarditis	MYH6 MYH7
Cardiac muscle contraction	MYH6 MYH7
Hypertrophic cardiomyopathy (HCM)	MYH6 MYH7
Dilated cardiomyopathy (DCM)	MYH6 MYH7
Ribosome	RPL7 RPL29
Adrenergic signaling in cardiomyocytes	MYH6 MYH7

Table S6: Genes identified in DMD-iCMs following 48 hr N-exo exposure – GO analysis gene list.

Click here to Download Table S6

Table S7: Genes identified in DMD-iCMs following 48 hr N-exo exposure – KEGG analysis gene list.

Click here to Download Table S7

Table S8: Genes identified in DMD-iCMs following 48 hr DMD-exo exposure – GO analysis gene list.

Click here to Download Table S8

Table S9: Genes identified in DMD-iCMs following 48 hr DMD-exo exposure – KEGG analysis gene list.

Click here to Download Table S9
